# Varying-Coefficient Additive Models with Density Responses and Functional Auto-Regressive Error Process

**DOI:** 10.3390/e27080882

**Published:** 2025-08-20

**Authors:** Zixuan Han, Tao Li, Jinhong You, Narayanaswamy Balakrishnan

**Affiliations:** 1 Division of Public Health Sciences, Fred Hutchinson Cancer Center, Seattle, WA 98109, USA; h_zxuan@163.com; 2 School of Statistics and Data Science, Shanghai University of Finance and Economics, Shanghai 200433, China; johnyou07@163.com; 3 Department of Mathematics and Statistics, McMaster University, Hamilton, ON L8S 4L8, Canada; bala@mcmaster.ca

**Keywords:** varying-coefficient, density response, functional auto-regressive error process, log-quantile density transformation

## Abstract

In many practical applications, data collected over time often exhibit autocorrelation, which, if unaccounted for, can lead to biased or misleading statistical inferences. To address this issue, we propose a varying-coefficient additive model for density-valued responses, incorporating a functional auto-regressive (FAR) error process to capture serial dependence. Our estimation procedure consists of three main steps, utilizing spline-based methods after mapping density functions into a linear space via the log-quantile density transformation. First, we obtain initial estimates of the bivariate varying-coefficient functions using a B-spline series approximation. Second, we estimate the error process from the residuals using spline smoothing techniques. Finally, we refine the estimates of the additive components by adjusting for the estimated error process. We establish theoretical properties of the proposed method, including convergence rates and asymptotic behavior. The effectiveness of our approach is further demonstrated through simulation studies and applications to real-world data.

## 1. Introduction

**Figure 1:**
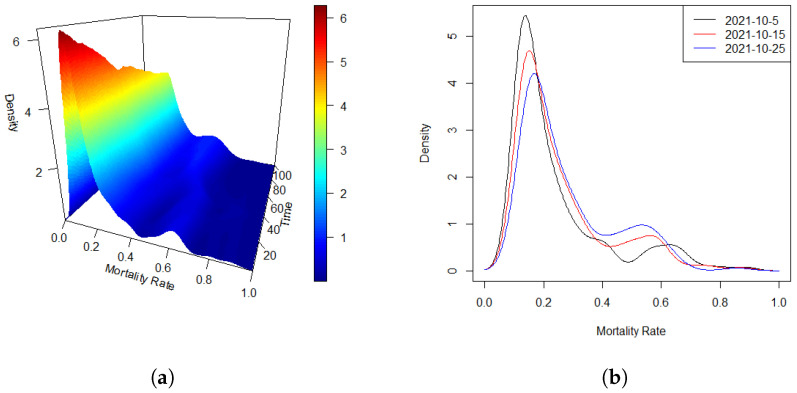
Densities of global COVID-19 mortality rates (‰) observed over a 100-day period. (**a**) Three-dimensional representation of the evolving density time series across the entire time span. (**b**) Density curves plotted for three selected days.

Density data or, more broadly, distributional data, are increasingly encountered across a wide range of scientific and applied research domains. Notable examples include the distributions of cross-sectional or intraday stock returns [1,2], mortality rate densities [3], and the distributions of intrahub connectivity in neuroimaging studies [4,5]. In many settings, such density functions are observed sequentially over time, forming what we refer to in this paper as a density time series. A motivating example is presented in Figure 1. Panel (a) displays a density time series of the global COVID-19 mortality rate (‰) over a 100-day period from 22 January 2020 to 15 April 2021. Panel (b) offers a complementary view by plotting the densities on three selected days, highlighting the temporal evolution of the distributional patterns. In this work, we study a regression framework in which the response consists of a density time series, while the predictors are scalar covariates. This setting allows for the exploration of how scalar factors influence the dynamic evolution of entire distributions over time.

Unlike conventional functional data, density functions do not form a linear space due to inherent constraints, such as nonnegativity and the requirement that they integrate with one. These restrictions pose significant challenges for directly applying standard functional data analysis techniques to random densities. To address this, several approaches have been proposed, which can be broadly grouped into two categories. The first approach involves transforming densities into a Hilbert space through suitable continuous and invertible mappings, thereby overcoming the nonlinear structure of the density space. For example, Petersen and Müller [6] introduced two such transformations, the log-hazard transformation and the log-quantile density (LQD) transformation, that map probability densities to an unrestricted space of square-integrable functions. Building on this, Han et al. [7] employed the LQD transformation to model density responses within an additive functional-to-scalar regression framework. Similarly, Kokoszka et al. [1] developed two methods for forecasting density functions derived from cross-sectional and intraday financial returns, using compositional data analysis and a modified log-quantile transformation combined with functional principal component (FPC) analysis and exponential smoothing techniques. The second category of methods takes a geometric perspective by defining appropriate metrics on the space of probability distributions. For instance, Talská et al. [8] used an infinite-dimensional extension of Aitchison geometry to construct a density-on-scalar linear regression model within Bayes-Hilbert spaces. Meanwhile, Petersen and Müller [3] studied Fréchet regression in general metric spaces equipped with the Wasserstein metric. Extending this line of work, Chen et al. [9] leveraged the geometry of tangent bundles in Wasserstein space to propose distribution-on-distribution regression models and developed auto-regressive extensions for distribution-valued time series. Additionally, Zhang et al. [10] explored auto-regressive models of order *p* for density-valued time series using the Wasserstein metric through a different methodological framework.

Let F denote the space of density functions *f* defined on a common support U. Without of generality, we assume that U=[0,1]. Given a transformation Ψ:F→L2, the conditional Fréchet mean of a random density *f*, given a covariate $X\in R^{d}$, is defined as

$$\mu \left(\cdot |X\right)=\arg\min_{d\in F}E(||\Psi \left(f\right)-{\Psi \left(d\right)||}_{2}^{2})X),$$

where the expectation *E* represents the joint distribution of (X,f).

This is equivalent to the following formulation:

Ψ(μ(·|X))(u)=E(Ψ(f)(u)|X),0≤u≤1,

leading to the fact that

$$\mu \left(s|X\right)=\Psi^{-1}(E(\Psi (f)(u)|X))\left(s\right),\;0\leq s\leq 1.$$

The data considered in this article consist of a density time series $d_{t}$, observed sequentially over time, along with associated with scalar predictors $\left(X_{t},Z_{t}\right)$. To facilitate the analysis of density functions, we employ the LQD transformation $\Psi :F\;\to \;L_{2}$, where F denotes the space of density functions *d* satisfying the moment condition $\int_{R}u^{2}d\left(u\right)du<\infty$. For each $d_{t}\in F$, let $F_{t}\left(y\right)$ be the corresponding cumulative distribution function with support on [0,1], and let $Q_{t}\left(u\right)$ denote the associated quantile function. The quantile density function is given by $q_{t}\left(u\right)$, i.e., $q_{t}\left(u\right)=Q_{t}^{\prime }\left(u\right)=\frac{d}{du}F_{t}^{-1}\left(u\right)$ for u∈[0,1]. Then, the LQD transformation of $d_{t}$ is defined as

$$\Psi \left(d_{t}\right)\left(u\right)=\log\left(\frac{d}{du}F_{t}^{-1}\left(u\right)\right),\;u\in \left[0,1\right].$$

In this study, we propose a varying-coefficient additive model with a functional auto-regressive error process to estimate the conditional expectation $E(\Psi \left(d_{t}\right)|X_{t},Z_{t})$. Under this framework, the density function $d_{t}$ can be expressed as

$$d_{t}=\Psi^{-1}\left(E\left(\Psi \left(d_{t}\right)|X_{t},Z_{t}\right)\right)+\delta_{t1},$$

where $\delta_{t1}$ represents the regression error.

In addition to $\delta_{t1}$, a second source of error commonly arises from the estimation of the density function $d_{t}$. Specifically, in most practical settings, the density $d_{t}$ is not directly observed. Instead, only a finite sample $Y_{t1},\cdots ,Y_{tn_{t}}∼d_{t}$ is available at each time point *t*, leading to an estimated density ${\hat{d}}_{t}$ given by

$${\hat{d}}_{t}=d_{t}+\delta_{t2},$$

where $\delta_{t2}$ denotes the error due to density estimation. Throughout this article, we assume that the sample size $n_{t}=n$ is fixed across time.

Following the approach of [6], we estimate $d_{t}$ using a modified kernel density estimator that addresses boundary effects. The estimator is defined as

$${\hat{d}}_{t}\left(y\right)=\sum_{i=1}^{n}K\left(\frac{y-Y_{ti}}{h}\right)w\left(y,h\right)/\sum_{i=1}^{n}\int_{0}^{1}K\left(\frac{s-Y_{ti}}{h}\right)w\left(s,h\right)ds,$$

where K is a symmetric kernel function with bandwidth h<1/2 and the weight function w(y,h) is designed to correct for boundary bias. Specifically, w(y,h) is given by

$$w\left(y,h\right)={\left(\int_{-y/h}^{1}K\left(u\right)du\right)}^{-1}I_{y\in [0,h)}+{\left(\int_{-1}^{(1-y)/h}K\left(u\right)du\right)}^{-1}I_{y\in (1-h,1]}+I_{y\in [h,1-h]}.$$

We assume that the kernel K is of bounded variation, symmetric about 0, and satisfies the following conditions: $\int_{0}^{1}K\left(u\right)du>0$; $\int_{R}\left|u\right|K\left(u\right)du$, $\int_{R}K^{2}\left(u\right)du$, and $\int_{R}\left|u\right|K^{2}\left(u\right)du$ are finite. Therefore, when fitting the regression model using the estimated density ${\hat{d}}_{t}$ in place of the true $d_{t}$, the model can be written as

$${\hat{d}}_{t}=\Psi^{-1}\left(E\left(\Psi \left(d_{t}\right)|X_{t},Z_{t}\right)\right)+\delta_{t1}+\delta_{t2}.$$

The key contribution of this article lies in the integration of density time series modeling with a functional auto-regressive (FAR) error process, a direction that has not been previously studied in the literature. A common assumption in regression analysis, including functional and density-based models, is the independence of random errors; however, this assumption is often violated in time-indexed data, where observations naturally exhibit serial dependence. By explicitly incorporating a FAR(1) structure into the error process, our approach effectively captures the temporal correlation inherent in density time series, thereby enhancing both the flexibility and accuracy of the model. Many real-world phenomena are characterized by time-evolving densities that exhibit strong temporal dependencies [11,12,13,14]. In these settings, the observed distribution at a given time point is not independent of previous distributions, but rather influenced by them through complex temporal dynamics. This phenomenon, commonly referred to as sequence dependence, cannot be adequately modeled under the assumption of independent errors. Ignoring such dependencies often leads to biased estimation, underestimated variability, and invalid inference, as documented in numerous empirical and theoretical studies. While FAR models have been widely explored as standalone tools for modeling functional time series data [11,12,13,14,15,16,17], their use as an error structure within a regression model for density-valued responses remains largely underdeveloped. This article addresses this methodological gap by embedding a FAR(1) process into the error term of a varying-coefficient additive regression framework tailored to density time series. This novel integration enables the model to more faithfully capture both the structured signal and the dynamic residual behavior present in such data. In addition to this modeling innovation, we make several theoretical contributions. Specifically, we develop a new estimation procedure that accommodates both the infinite-dimensional nature of the response and the temporal dependence in the errors. We further derive the asymptotic normality of the proposed estimator, which requires nontrivial extensions of existing techniques in functional data analysis and Hilbert space theory. This allows for valid statistical inference and construction of confidence intervals in practice. In summary, this work contributes a new class of models for density-valued time series with auto-regressive error dynamics, bridging gaps between functional time series, density regression, and auto-regressive modeling. The proposed framework provides both a theoretical foundation and a practical tool for analyzing complex time-evolving distributional data in a wide range of applications.

The remainder of this article is organized as follows: Section 2 presents the methodology for constructing a varying-coefficient additive model with a density response, incorporating a functional auto-regressive (FAR) error process. In Section 3, we propose a three-step estimation procedure for the bivariate varying-coefficient components within the model. Section 4 establishes the theoretical properties of the proposed model and discusses related inferential results. Section 5 reports Monte Carlo simulation studies that evaluate the efficiency and robustness of our approach. In Section 6, we demonstrate the practical utility of the model through applications to COVID-19 mortality data and U.S. income distribution data. Finally, Section 7 offers concluding remarks, and the Supplementary Material contains detailed proofs of the theoretical results.

## 2. Model Setup

In this article, we focus on modeling density responses. Due to the inherent constraints of density functions, namely nonnegativity and integration to one, we work with their representations after applying the log-quantile density (LQD) transformation.

Our primary goal is to estimate the conditional expectation $E(\Psi \left(d_{t}\right)\left(u\right)|x)$ through the transformation of density function, expressed as

(1) $$E\left(\Psi \left(d_{t}\right)\left(u\right)|x\right)=\sum_{m=1}^{k}z_{t,m}g_{m}\left(u,x_{t,m}\right),\;0\leq u\leq 1,$$

which leads to the proposed varying-coefficient additive models with density responses and functional auto-regressive error process FAR(p) (DVCA-FAR):

(2) $$f_{t}\left(u\right)=\Psi \left(d_{t}\right)\left(u\right)=\sum_{m=1}^{k}z_{t,m}g_{m}\left(u,x_{t,m}\right)+\varepsilon_{t}\left(u\right),\;0\leq u\leq 1,\;1\leq t\leq T,$$

where the error process $\varepsilon_{t}\left(u\right)$ follows a functional auto-regressive process of order *p*:

(3) $$\varepsilon_{t}\left(u\right)=\int \gamma_{1}\left(s,u\right)\varepsilon_{t-1}\left(s\right)ds+\cdots +\int \gamma_{p}\left(s,u\right)\varepsilon_{t-p}\left(s\right)ds+e_{t}\left(u\right).$$

In this framework, the random density $d_{t}\left(\cdot \right)\in F$ serves as the response variable, and $\Psi :F⟶L_{2}$ denotes the LQD transformation. Each density is associated with two sets of k-dimensional covariates, $x_{t}={\left(x_{t,1},\cdots ,x_{t,k}\right)}^{\tau }$ and $z_{t}={\left(z_{t,1},\cdots ,z_{t,k}\right)}^{\tau }$, with supports $S_{x}$ and $S_{z}$, respectively. Without loss of generality, we assume $S_{x}=S_{z}=\left[0,1\right]$. In this article, the covariate $x_{t}$ can represent $z_{t}$ or the rescaled time index t/T.

The bivariate functions $g_{m}\left(\cdot ,x_{m}\right)$ capture the effects of the covariates z, while the kernel functions $\gamma_{l}\left(\cdot ,\cdot \right)$ are smooth and satisfy the integrability condition $\;\int \int \;\gamma_{l}^{2}\left(s,u\right)duds<\infty$. The innovation process $e_{t}\left(u\right)$ consists of independent and identically distributed random functions with zero mean $E(e_{t}\left(u\right))=0$ and covariance function $Cov\left(e_{t}\left(u\right),e_{t}\left(s\right)\right)=\sigma_{t}^{2}\left(u,s\right)$.

When the density functions are estimated, denoted by $\hat{d}$, we write ${\hat{f}}_{t}=\Psi \left({\hat{d}}_{t}\right)$. Then DVCA-FAR model then takes the form

(4) $${\hat{f}}_{t}\left(u\right)=\sum_{m=1}^{k}z_{t,m}g_{m}\left(u,x_{t,m}\right)+\varepsilon_{t}\left(u\right)+\varepsilon_{f_{t}}\left(u\right),\;0\leq u\leq 1,$$

where $\varepsilon_{f_{t}}\left(u\right)$ represents the additional random error introduced by the transformation of estimated density.

## 3. Three-Step Estimation Methodology

We propose a three-step estimation procedure to estimate the varying-coefficient functions in the presence of a functional auto-regressive error structure. In the first step, we apply B-spline smoothing to obtain initial estimates of the bivariate varying-coefficient functions, ignoring the temporal dependence in the error process. In the second step, using the initial estimates and the transformed response, we estimate the error component. Specifically, the order and structure of the functional auto-regressive (FAR) process are determined using the sequential testing procedure proposed by Kokoszka and Reimherr [16]. In the final step, after removing the estimated FAR error from the response, we refine the estimation of the varying-coefficient functions using the spline-based method to obtain improved results.

### 3.1. Initial Estimation of Bivariate Varying-Coefficient Function

To begin, we estimate the bivariate varying-coefficient functions $g_{m}\left(u,x_{m}\right)$, for m=1,⋯,k, by applying a tensor product B-spline approximation, ignoring the temporal structure in the error term.

Let $\left\{B_{0}\left(u\right),\cdots ,B_{N_{0}}\left(u\right)\right\}$ denote a set of B-spline basis functions of order *q* with $L_{0}$ interior knots, defined on the domain of u∈[0,1], so that $N_{0}+1=L_{0}+q$. Similarly, for each m=1,⋯,k, let $\left\{B_{0,m}\left(x_{m}\right),\cdots ,B_{N_{m},m}\left(x_{m}\right)\right\}$ be a set of B-spline basis functions of order *q* for the covariate $x_{m}$, with $L_{m}$ interior knots, so that $N_{m}+1=L_{m}+q$. Let $b_{j,m}^{*}\left(x_{m}\right)$ denote the normalized B-spline basis functions of $B_{j,m}\left(x_{m}\right)$ for $x_{m}$, and define the scaled basis $b_{r}\left(u\right)=N_{0}^{1/2}B_{r}\left(u\right)$ of $B_{r}\left(u\right)$.

The tensor product of the B-spline basis functions is given by

$$b_{r,j,m}\left(u,x_{m}\right)=b_{r}\left(u\right)b_{j,m}^{*}\left(x_{m}\right),\;1\leq r\leq N_{0},\;1\leq j\leq N_{m},\;1\leq m\leq k.$$

Using this basis, the function of $g_{m}\left(u,x_{m}\right)$ can be approximated as

$$g_{m}\left(u,x_{m}\right)\approx \sum_{r=1}^{N_{0}}\sum_{j=1}^{N_{m}}\lambda_{r,j,m}b_{r,j,m}\left(u,x_{m}\right),\;1\leq m\leq k,$$

where $\lambda_{r,j,m}$ are the spline coefficients.

The least squares estimator of $g_{m}\left(u,x_{m}\right)$ is then given by

(5) $${\tilde{g}}_{m}\left(u,x_{m}\right)=\sum_{r=1}^{N_{0}}\sum_{j=1}^{N_{m}}{\tilde{\lambda }}_{r,j,m}b_{r,j,m}\left(u,x_{m}\right),\;1\leq m\leq k,$$

where the vector of estimated coefficients is defined as $\tilde{\lambda }={\left({\tilde{\lambda }}_{1,1,1},\cdots ,{\tilde{\lambda }}_{N_{0},N_{k},k}\right)}^{\tau }$, a $\left(N_{0}\sum_{m=1}^{k}N_{m}\right)$-dimensional parameter vector obtained by solving

(6) $$\tilde{\lambda }=\arg\min_{\lambda }\sum_{t=1}^{T}\sum_{i=1}^{n}{\left[{\hat{f}}_{t}\left(u_{i}\right)-\sum_{m=1}^{k}z_{t,m}\sum_{r=1}^{N_{0}}\sum_{j=1}^{N_{m}}\lambda_{r,j,m}b_{r,j,m}\left(u_{i},x_{t,m}\right)\right]}^{2}.$$

Theoretical properties of this estimator are established in Theorem 1, which shows that the initial estimators ${\tilde{g}}_{m}\left(u,x_{m}\right)$ are uniformly consistent under suitable regularity conditions.

### 3.2. Estimation of FAR Error Process

With the initial estimates ${\tilde{g}}_{m}\left(u,x_{m}\right)$ obtained, we proceed to estimate the FAR error process. To do so, define the residuals as

(7) $${\tilde{\varepsilon }}_{t}\left(u\right)=f_{t}\left(u\right)-\sum_{m=1}^{k}z_{t,m}{\tilde{g}}_{m}\left(u,x_{t,m}\right),\;1\leq t\leq T.$$

Let $\rho_{t}\left(u\right)=\sum_{l=1}^{p}\int \gamma_{l}\left(s,u\right)\varepsilon_{t-l}\left(s\right)ds$ denote the additive component in the FAR(p) error process (3). Then, the FAR process can be written as

$$\varepsilon_{t}\left(u\right)=\rho_{t}\left(u\right)+e_{t}\left(u\right),$$

where $e_{t}\left(u\right)$ is a zero-mean innovation term.

Let $\left\{B_{0}\left(u\right),B_{2}\left(u\right),\cdots ,B_{N}\left(u\right)\right\}$ be a set of B-spline basis functions of order *q* with *L* interior knots, such that N+1=L+q. Define the tensor product of the B-spline basis as

$$b_{r,j}\left(u,s\right)=B_{r}\left(u\right)B_{j}\left(s\right),\;1\leq r,j\leq N.$$

Using this basis, the FAR kernel functions $\gamma_{l}\left(\cdot ,\cdot \right)$ are approximated as

$$\gamma_{l}\left(s,u\right)=\sum_{r=1}^{N}\sum_{j=1}^{N}\mu_{r,j,l}b_{r,j}\left(u,s\right),\;1\leq l\leq p.$$

The vector of spline coefficients $\mu ={\left(\mu_{1,1,1},\cdots ,\mu_{N,N,p}\right)}^{\tau }\in R^{pN^{2}}$ is obtained by minimizing the following squared error criterion:

$$\hat{\mu }=\arg\min_{\mu }\sum_{t=p+1}^{T}\sum_{i=1}^{n}{\left[{\tilde{\varepsilon }}_{t}\left(u_{i}\right)-\sum_{l=1}^{p}\sum_{r=1}^{N}\sum_{j=1}^{N}\mu_{r,j,l}\int b_{r,j}\left(u_{i},s\right){\tilde{\varepsilon }}_{t-l}\left(s\right)ds\right]}^{2}.$$

The estimated FAR kernel functions $\gamma_{l}\left(\cdot ,\cdot \right)$ and the additive component of the error process $\rho_{t}\left(u\right)$ are given by

$${\hat{\gamma }}_{l}\left(s,u\right)=\sum_{r=1}^{N}\sum_{j=1}^{N}{\hat{\mu }}_{r,j,l}b_{r,j}\left(u,s\right),\;1\leq l\leq p,$$

and

(8) $${\hat{\rho }}_{t}\left(u\right)=\sum_{l=1}^{p}\sum_{r=1}^{N}\sum_{j=1}^{N}{\hat{\mu }}_{r,j,l}\int b_{r,j}\left(u,s\right){\hat{\varepsilon }}_{t-l}\left(s\right)ds,\;0\leq u\leq 1,\;p+1\leq t\leq T,$$

respectively.

Since the order *p* of the FAR error process is typically unknown in practice, we employ the sequential testing procedure proposed by [16] to determine the optimal order *p*. The details of this procedure are provided in Section 3.4.2.

### 3.3. Improved Estimation of Bivariate Varying-Coefficient Function

With an estimate of the FAR error component obtained, we now refine the estimation of the varying-coefficient functions $g_{m}\left(u,x_{m}\right)$ by removing the estimated serial dependence (8) from the response $f_{t}\left(u\right)$.

Define the adjusted response function as

$$f_{t}^{c}\left(u\right)=f_{t}\left(u\right)-\sum_{l=1}^{p}\int \gamma_{l}\left(s,u\right)\varepsilon_{t-l}\left(s\right)ds,\;0\leq u\leq 1,\;p+1\leq t\leq T,$$

and its empirical estimates $f_{t}^{c}\left(u\right)$ as

$${\hat{f}}_{t}^{c}\left(u\right)=f_{t}\left(u\right)-\sum_{l=1}^{p}\int {\hat{\gamma }}_{l}\left(s,u\right){\hat{\varepsilon }}_{t-l}\left(s\right)ds,\;0\leq u\leq 1,\;p+1\leq t\leq T.$$

From the model specification, we have

$$f_{t}^{c}\left(u\right)=\sum_{m=1}^{k}z_{t,m}g_{m}\left(u,x_{t,m}\right)+e_{t}\left(u\right),\;0\leq u\leq 1.$$

which allows us to re-estimate $g_{m}\left(u,x_{m}\right)$ by repeating the same spline-based procedure as described in Section 3.1, but now applied to the corrected responses ${\hat{f}}_{t}^{c}\left(u\right)$.

The improved spline approximation estimates take the form

(9) $${\hat{g}}_{m}\left(u,x_{m}\right)=\sum_{r=1}^{N_{0}}\sum_{j=1}^{N_{m}}{\hat{\lambda }}_{r,j,m}b_{r,j,m}\left(u,x_{m}\right),\;1\leq m\leq k,$$

where the coefficient vector $\hat{\lambda }={\left({\hat{\lambda }}_{1,1,1},\cdots ,{\hat{\lambda }}_{N_{0},N_{k},k}\right)}^{T}$ is a $\left(N_{0}\sum_{m=1}^{k}N_{m}\right)$-dimensional vector obtained by minimizing

(10) $$\hat{\lambda }=\arg\min_{\lambda }\sum_{t=1}^{T}\sum_{i=1}^{n}{\left[{\hat{f}}_{t}^{c}\left(u_{i}\right)-\sum_{m=1}^{k}z_{t,m}\sum_{r=1}^{N_{0}}\sum_{j=1}^{N_{m}}\lambda_{r,j,m}b_{r,j,m}\left(u_{i},x_{t,m}\right)\right]}^{2}.$$

Theoretical guarantees for this refined estimator are provided in Theorems 2 and 3, which establish its uniform convergence and asymptotic normality under regularity conditions. In addition, simulation results reported in Section 5 demonstrate that the improved estimator ${\hat{g}}_{m}\left(u,x_{m}\right)$ achieves greater efficiency and accuracy compared to the initial estimator ${\tilde{g}}_{m}\left(u,x_{m}\right)$.

### 3.4. Implementation

#### 3.4.1. Selection of Bandwidth

In empirical applications, it is necessary to estimate the underlying density functions before model fitting. This step requires selecting an appropriate bandwidth for the modified kernel density estimator. In this section, we adopt a leave-one-out cross-validation (LOOCV) strategy to select the optimal bandwidth.

Specifically, the bandwidth *h* is chosen to minimize the following mean squared error (MSE) criterion:

$$CV\left(h\right)=\frac{1}{nT}\sum_{t=1}^{T}\sum_{i=1}^{n}{\left[d_{t}\left(y_{ti}\right)-{\hat{d}}_{t}^{\left(-i\right)}\left(y_{ti}\right)\right]}^{2},$$

where for each i=1,⋯,n, ${\hat{d}}_{t}^{\left(-i\right)}\left(y_{ti}\right)$ denotes the density estimate of $d_{t}\left(y_{ti}\right)$ with bandwidth *h* at point $y_{ti}$ using all observations from time point *t* except the *i*-th one.

#### 3.4.2. Identifying the Order of the FAR Process

To determine the order *p* of FAR error process, we apply the sequential testing procedure procedure proposed by [16]. The method frames FAR modeling as a fully functional linear regression with dependent regressors and systematically tests whether increasing the order improves the model fit.

The procedure tests the following nested hypotheses:

$$H_{0,p}:\left\{\varepsilon_{t}\right\}\;\mathrm{follows}\;a\;FAR\left(p\right)\;\mathrm{vs}\;H_{a,p+1}:\left\{\varepsilon_{t}\right\}\;\mathrm{follows}\;a\;FAR\left(p+1\right),\;p=0,1,2,\cdots ,$$

Here, FAR(0) corresponds to an independent and identically distributed process. The testing begins at p=0 and continues sequentially. The process stops when $H_{0,p}$ is not rejected, at which point the selected model order is taken to be *p*. See [16] for the full theoretical development.

To construct the test statistic, define the following components. Let

$$\eta_{j}\left(s\right)=\sum_{l=1}^{p}{\tilde{\varepsilon }}_{j-l}\left(sp-\left(l-1\right)\right)I_{l}\left(s\right),\;\varphi \left(s,u\right)=p\sum_{l=1}^{p}\gamma_{l}\left(sp-\left(l-1\right),u\right)I_{l}\left(s\right),$$

where $I_{l}$ is the indicator function on the interval [(l−1)/p,l/p]. Denote

$${\hat{C}}_{\eta }\left(s,u\right)=\frac{1}{T}\sum_{j=1}^{T}\left(\eta_{j}\left(s\right)-\bar{\eta }\left(s\right)\right)\left(\eta_{j}\left(u\right)-\bar{\eta }\left(u\right)\right)$$

as the empirical covariance operator of $\left\{\eta_{j}\right\}$, where $\bar{\eta }\left(s\right)$ is the sample mean function. Let $\left\{{\hat{x}}_{j}\right\}$ and $\left\{{\hat{\lambda }}_{j}\right\}$ be the eigenfunctions and corresponding decreasingly ordered eigenvalues of ${\hat{C}}_{\eta }$, respectively. We retain only the first $q_{\eta }$ eigenfunctions for dimensionality reduction. Similarly, for the functional responses $\left\{\pi_{j}\right\}$, define eigenpairs $\left\{{\hat{y}}_{j}\right\}$ and corresponding number $q_{\pi }$ analogously.

For the product space $L^{2}\left(\left[0,1\right]\times \left[0,1\right]\right)$, define the projections

$$\eta \left(j,k\right)=\langle \eta_{j},{\hat{x}}_{k}\rangle ,\;\pi \left(j,m\right)=\langle \pi_{j},{\hat{y}}_{m}\rangle ,\;\psi \left(k,m\right)=\langle \varphi ,{\hat{x}}_{k}⊗{\hat{y}}_{m}\rangle .$$

Denote the matrices $\eta ={\left[\eta \left(j,k\right)\right]}_{T\times q_{\eta }},\pi ={\left[\pi \left(j,m\right)\right]}_{T\times q_{\pi }}$, and $\psi ={\left[\psi \left(k,m\right)\right]}_{q_{\eta }\times q_{\pi }}$, j=1,⋯,T, $k=1,\cdots ,q_{\eta }$, $m=1,\cdots ,q_{\pi }$.

Next, construct the matrix $\hat{A}\in R^{q_{\eta }\times q_{\eta }}$ with entries

$$\hat{A}\left(k,k^{\prime }\right)=\langle {\hat{x}}_{k,p},{\hat{x}}_{k^{\prime },p}\rangle ,\;\mathrm{where}\;{\hat{x}}_{k,p}\left(s\right)={\hat{x}}_{k}\left(\frac{s+p-1}{p}\right),\;0\leq s\leq 1.$$

Define the orthonormal eigenvectors ${\hat{\beta }}_{k}$ with corresponding ordered eigenvalues ${\hat{\xi }}_{1}\geq \cdots \geq {\hat{\xi }}_{q_{\eta }}$ as $\hat{A}{\hat{\beta }}_{k}={\hat{\xi }}_{k}{\hat{\beta }}_{k},\;1\leq k\leq q_{\eta }$. Define the matrix $\hat{B}=\left[{\hat{\beta }}_{1},\cdots ,{\hat{\beta }}_{q^{*}}\right]$, where

$$q^{*}=\max\{k\in \left\{1,\cdots ,q_{\eta }\right\}:||{\hat{z}}_{k,p}{\left|\right|}^{2}\geq 0.9p\},\;\mathrm{with}\;{\hat{z}}_{k,p}\left(s\right)=\sum_{i=1}^{q_{\eta }}{\hat{\beta }}_{k,i}{\hat{x}}_{k,p}\left(s\right).$$

Finally, following [16], the test statistic is constructed as

(11) $${\hat{\tau }}_{p}=\frac{1}{T}{\left(vec[{\hat{B}}^{\tau }\hat{\psi }]\right)}^{\tau }{\left[\left(I_{q_{\varepsilon }}⊗{\hat{B}}^{\tau }\right)\left(\hat{C}⊗\hat{\Lambda }\right)\left(I_{q_{\varepsilon }}⊗\hat{B}\right)\right]}^{-1}vec\left[{\hat{B}}^{\tau }\hat{\psi }\right],$$

where $\hat{\Lambda }=diag\left({\hat{\lambda }}_{1},\cdots ,{\hat{\lambda }}_{q_{\eta }}\right),\;\hat{C}=\frac{1}{T}{\left(\pi -\eta \hat{\psi }\right)}^{\tau }\left(\pi -\eta \hat{\psi }\right)$. Under $H_{0,p}$, the test statistic ${\hat{\tau }}_{p+1}$ asymptotically follows a chi-squared distribution with degrees of freedom $q_{\pi }q^{*}$.

## 4. Theoretical Results

In this section, we investigate the asymptotic properties of both the initial and improved estimators of the bivariate varying-coefficient functions $g_{m}\left(u,x_{m}\right)$. We also establish the consistency of the estimator for the order *p* of the functional auto-regressive (FAR) process. All technical proofs are deferred to the Supplementary Materials.

Throughout the remainder of this paper, for any fixed interval [a,b], we denote the space of functions that are *l*-times continuously differentiable on [a,b] as $C^{\left(l\right)}\left[a,b\right]=\left\{g|g^{\left(l\right)}\in \left[a,b\right]\right\}$. Let $Lip\left(\left[a,b\right],C\right)=\{g||g\left(x\right)-g\left(x^{\prime }\right)|\leq |x-x^{\prime }|,\forall x,x^{\prime }\in \left[a,b\right]\}$ denote the class of Lipschitz-continuous functions with Lipschitz constant C>0. Let $S_{x_{m}}$ and $S_{z_{m}}$ denote the supports of $x_{m}$ and $z_{m}$, respectively. Then, the supports of the covariate vectors x and z are given by $S_{x}=\prod_{m=1}^{k}S_{x_{m}}$ and $S_{z}=\prod_{m=1}^{k}S_{z_{m}}$, respectively. The following regularity conditions are imposed to derive the asymptotic properties of the proposed estimators.

(A1) For any density function d∈F, *d* is differentiable and there exists a constant M>1 such that ${\left||d|\right|}_{\infty }$, $||1/{d||}_{\infty }$, and $\left|\right|d^{\prime }{\left|\right|}_{\infty }$ are all bounded by *M*.
(A2) (a) The kernel function K is Lipschitz-continuous, bounded, and symmetric about zero. Furthermore, $K\in Lip(\left[-1,1\right],L_{k})$ for some constant $L_{k}>0$. (b) The kernel function satisfies the following conditions: $\int_{0}^{1}K\left(u\right)du>0$, $\int_{R}\left|u\right|K\left(u\right)du<\infty$, $\int_{R}K^{2}\left(u\right)du<\infty$, and $\int_{R}\left|u\right|K^{2}\left(u\right)du<\infty$.
(A3) The covariates $x_{t,m},z_{t,m}$, for 1≤m≤k, and the error functions $\varepsilon_{t}\left(u\right)$ satisfy the following moment conditions: for some s>2,
  $$\max_{1\leq t\leq T}\max_{1\leq m\leq k}E(|x_{t,m}{|}^{2s})<\infty ,\max_{1\leq t\leq T}\max_{1\leq m\leq k}E(|z_{t,m}{|}^{2s})<\infty ,\max_{1\leq t\leq T}\underset{u}{\sup}E(|\varepsilon_{t}\left(u\right){|}^{2s})<\infty .$$
  For each t=1,⋯,T, the covariance function of the error process $Cov\left(\varepsilon_{t}\left(s\right),\varepsilon_{t}\left(v\right)\right)=\Sigma_{t}\left(s,v\right)$ has finite nondecreasing eigenvalues $\lambda_{1}\leq \cdots \leq \lambda_{max}$ such that $\sum_{j}\lambda_{j}<\infty$.
(A4) The varying-coefficient functions $g_{m}\left(u,x_{m}\right)$ are continuous over the domain $\left[0,1\right]\times \left[a_{m},b_{m}\right]$ and are twice continuously partially differentiable with respect to *u* and $x_{m}$, for each 1≤m≤k. Here, $\left[a_{m},b_{m}\right]$ is a compact subset of the support $S_{x_{m}}$.
(A5) The numbers of basis functions satisfy $N_{0}∼{\left(nT\right)}^{1/6}\log nT$, $N_{m}∼{\left(nT\right)}^{1/6}\log nT$, 1≤m≤k, and the bandwidth satisfies $h∼n^{-1/3}$, as n,T→∞.

**Remark 1.**  *Assumption (A1) is standard and ensures the well-posedness of density transformations. Assumption (A2) imposes mild conditions on the kernel function K(·), which are satisfied by commonly used kernel functions such as the uniform and Epanechnikov kernels. The moment conditions in (A3) are essential for establishing the uniform convergence and other asymptotic properties of spline-based estimators. Assumption (A4) requires only moderate smoothness of the coefficient functions and is relatively weak compared to traditional nonparametric assumptions. Lastly, the growth conditions in (A5) are widely adopted in the literature on spline smoothing to ensure optimal convergence rates.*

We begin by examining the uniform convergence of the initial estimator of bivariate functions $g_{m}\left(u,x_{m}\right)$, as stated in Theorem 1.

**Theorem 1.**  *Assume that Assumptions (A1)–(A5) hold, and let ${\tilde{g}}_{m}\left(u,x_{m}\right)$ denote the initial estimator of $g_{m}\left(u,x_{m}\right)$, defined in Equation (5), for m=1,⋯,k. Then, as n→∞ and T→∞, we have*

$$\underset{u,x_{m}\in \left[0,1\right]}{\sup}\left|{\tilde{g}}_{m}\left(u,x_{m}\right)-g_{m}\left(u,x_{m}\right)\right|=O_{p}\left({\left(nT\right)}^{-1/3}\log\left(nT\right)+n^{-1/3}\right).$$

Theorem 2 characterizes the uniform convergence of the improved estimation of $g_{m}\left(u,x_{m}\right)$, and Theorem 3 describes the asymptotic properties of both the initial and improved estimators.

**Theorem 2.**  *Assume that Assumptions (A1)–(A5) hold, and that the order p of the functional error process is known. Let ${\hat{g}}_{m}\left(u,x_{m}\right)$ denote the improved estimator of $g_{m}\left(u,x_{m}\right)$, as defined in Equation (9), for m=1,⋯,k. Then, as n→∞ and T→∞, it holds that*

$$\underset{u,x_{m}\in \left[0,1\right]}{\sup}\left|{\hat{g}}_{m}\left(u,x_{m}\right)-g_{m}\left(u,x_{m}\right)\right|=O_{p}({\left(nT\right)}^{-1/3}{\left(\log\left(nT\right)\right)}^{-2}+n^{-1/3}).$$

To establish the asymptotic normality of the estimators, we introduce the following notations. Denote $b\left(u,x_{t,m}\right)={\left(b_{1,1,m}\left(u,x_{t,m}\right),\cdots ,b_{N_{0},N_{m},m}\left(u,x_{t,m}\right)\right)}^{\tau }$, $b_{z}\left(u,x_{t,m}\right)=z_{t,m}b\left(u,x_{t,m}\right)$, ${\mathrm{Bz}}_{t,m}={\left(b_{z}\left(u_{1},x_{t,m}\right),\cdots ,b_{z}\left(u_{n},x_{t,m}\right)\right)}_{n\times N_{0}N_{m}}^{\tau }$, $B_{m}={\left({{\mathrm{Bz}}_{1,m}}^{\tau },\cdots ,{{\mathrm{Bz}}_{T,m}}^{\tau }\right)}^{\tau }$, $B=(B_{1},\cdots ,B_{k})$, and $B_{*}=B/\sqrt{nT}$.

Let $A_{m}=\left(0,\cdots ,I,\cdots ,0\right)$ denote a block matrix of dimension 1×k, where the *m*-th block is an identity matrix of size $N_{0}N_{m}\times N_{0}N_{m}$, and all other blocks are zero matrices of appropriate dimensions.

**Theorem 3.**  *Assume that Assumptions (A1)–(A5) hold, let ${\tilde{g}}_{m}\left(u,x_{m}\right)$ and ${\hat{g}}_{m}\left(u,x_{m}\right)$ denote the initial and improved estimators of $g_{m}\left(u,x_{m}\right)$, as defined in Equations (5) and (9), m=1,⋯,k, respectively. Then, as n≫T→∞, for any u∈(0,1) and $x_{m}\in \left[0,1\right]$, the following results hold:*

*(i)*  *The initial estimator ${\tilde{g}}_{m}\left(u,x_{m}\right)$ is asymptotically normally distributed, i.e.,*
$$\sqrt{nT}{\left(C_{m}\Sigma_{\varepsilon }C_{m}^{\tau }\right)}^{-1}\left({\tilde{g}}_{m}\left(u,x_{m}\right)-g_{m}\left(u,x_{m}\right)\right)\overset{D}{\to }N\left(0,1\right),$$
  where $C_{m}=b^{\tau }\left(u,x_{m}\right)E\left(A_{m}{\left(B_{*}^{\tau }B_{*}\right)}^{-1}B_{*}^{\tau }\right)$, the covariance matrix $\Sigma_{\varepsilon }={\left(\Sigma_{t,s}\right)}_{1\leq t,s\leq T}$, with $\Sigma_{t,s}=Cov\left(\varepsilon_{t},\varepsilon_{s}\right)$.
*(ii)*  *The improved estimator ${\hat{g}}_{m}\left(u,x_{m}\right)$ is asymptotically normally distributed, i.e.,*
$$\sqrt{nT}{\left(C_{m}\Xi_{\varepsilon }C_{m}^{\tau }\right)}^{-1}\left({\hat{g}}_{m}\left(u,x_{m}\right)-g_{m}\left(u,x_{m}\right)\right)\overset{D}{\to }N\left(0,1\right),$$
  *where the covariance matrix $\Xi_{\varepsilon }=\mathrm{diag}{\left(\Xi_{t,t}\right)}_{1\leq t\leq T}$, with $\Xi_{t,t}\left(u,s\right)=\sigma_{t}^{2}\left(u,s\right)$.*

## 5. Numerical Study

In this section, we present two simulation studies designed to evaluate the performance of the proposed identification and estimation procedures for the additive model.

### 5.1. Case 1

This scenario aims to assess the estimation accuracy of the proposed method when the order of the auto-regressive error process is known with finite *n* and *T*. We consider a DVCA-FAR(1) model given by

(12) $$f_{t}\left(u\right)=z_{t,1}g_{1}\left(u,x_{t,1}\right)+z_{t,2}g_{2}\left(u,x_{t,2}\right)+\varepsilon_{t}\left(u\right),\;0\leq u\leq 1,$$

and the functional error process $\varepsilon_{t}\left(u\right)$ takes form as

$$\varepsilon_{t}\left(u\right)=\int \gamma_{1}\left(s,u\right)\varepsilon_{t-1}\left(s\right)ds+e_{t}\left(u\right),\;2\leq t\leq T.$$

The bivariate varying-coefficient functions are specified as

$$g_{1}\left(u,x_{t,1}\right)=\sin\left(2\pi u\right)\left(2x_{t,1}-1\right),\;g_{2}\left(u,x_{t,2}\right)=\sin\left(2\pi u\right)\sin\left(2\pi x_{t,2}\right),$$

and the coefficient functions and innovation process are given by

$$\gamma_{1}\left(s,u\right)=0.2us,\;e_{t}\left(u\right)=0.2\eta_{t,1}\sin\left(\pi u\right)+\eta_{t,2}\sin\left(2\pi u\right),$$

with $\eta_{t,1}∼N\left(0,0.1^{2}\right)$, $\eta_{t,2}∼N\left(0,0.05^{2}\right)$, and $\eta_{t,1}$ are independent of $\eta_{t,2}$ for u∈[0,1].

The covariates are generated as follows: $z_{t,1}∼N\left(0,1\right),z_{t,2}∼N\left(0,0.5^{2}\right)$, and ${\left(x_{t,1},x_{t,2}\right)}^{\tau }={\left(\Phi \left(v_{t,1}\right),\Phi \left(v_{t,2}\right)\right)}^{\tau }$, 1≤t≤T, where Φ denotes the cumulative distribution function of the standard normal distribution and $v_{t,1}$, $v_{t,2}$ are independent standard normal variables.

To generate the response densities, for each given Z=z and X=x, let α(u,x,z) be the additive predictor given by $\alpha \left(u,x,z\right)=\sum_{m=1}^{p}z_{m}g_{m}\left(u,x_{m}\right)$. The conditional quantile function Q(·|x,z) with the error process ε(u), corresponding to the density $d_{t}$, is constructed as $Q\left(u|x,z\right)=F^{-1}\left(u|x,z\right)=\theta {\left(x,z\right)}^{-1}\int_{0}^{u}\exp\left\{\alpha \left(v,x,z\right)+\varepsilon \left(v\right)\right\}dv$, where $\theta \left(x,z\right)=\int_{0}^{1}\exp\left\{\alpha \left(v,x,z\right)+\varepsilon \left(v\right)\right\}dv$.

Given this construction, we generate response samples by applying the quantile function to uniform random variables $\left\{U_{t,1}\leq \cdots \leq U_{t,n_{t}}\right\}∼U\left(0,1\right)$, which are independent of $X_{t}$ and $Z_{t}$. Specifically, for each 1≤t≤T, we obtain the random samples $Y_{t}=\left\{Y_{t,j}=Q\left(U_{t,j}|X_{t},Z_{t}\right):1\leq j\leq n_{t}\right\}$, ensuring that $Y_{t,1},\cdots ,Y_{t,n_{t}}∼d_{t}$, where $d_{t}$ denotes the response density. The transformed density, as used in model (12), is then defined as $f_{t}\left(u\right)=\Psi \left(d_{t}\left(u\right)\right)$. For simplicity, we assume that independent and identically distributed observations are available for each response distribution, i.e., $n_{t}=n$.

**Figure 2:**
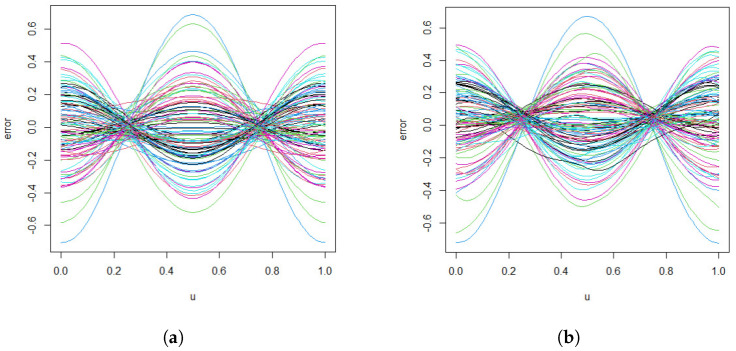
Average estimates of the FAR(1) error process ε(u) obtained from 200 Monte Carlo replications with sample size T=100 and n=100 observations. Panel (**a**) represents true curves, while panel (**b**) represents spline-based estimates. Each color represents a curve corresponding to an individual simulated subject.

**Figure 3:**
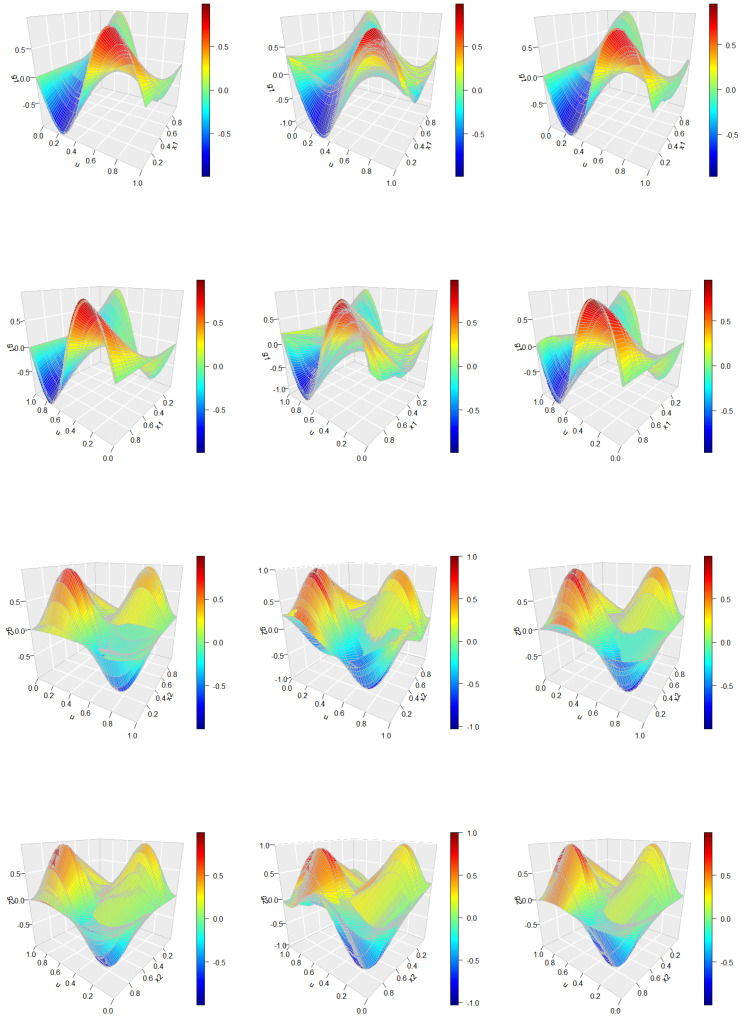
Average estimates of the bivariate functions $g_{m}\left(u,x_{m}\right),m=1,2$. **Left panels**: true density surfaces, **middle panels**: initial spline-based estimates, **right panels**: improved estimates after adjusting for the error structure. Top two panels correspond to $g_{1}\left(u,x_{1}\right)$ viewed from two different angles, bottom panels illustrate $g_{2}\left(u,x_{2}\right)$.

The simulation is conducted with T=100,n=100, and results are averaged over 200 Monte Carlo replications. Figure 2 presents the true error process ε(u) in panel (a) and its corresponding spline-based estimations in panel (b), demonstrating a high degree of accuracy in error recovery. Figure 3 provides a a comparative view of the bivariate function estimates. Specifically, the left panel displays the true surfaces of $g_{m}\left(u,x_{m}\right)$, while the middle and right panels show the average of the initial and improved estimations, respectively. The initial estimates are obtained without accounting for the FAR(1) error structure, whereas the improved estimates incorporate the estimated error process. To facilitate visual comparison, the surfaces are presented from two distinct viewing angles. This allows for a more comprehensive assessment of the estimation performance before and after error correction.

As illustrated in Figure 2, the proposed method achieves a highly accurate estimation of the error process. Moreover, the right panel of Figure 3 clearly demonstrates that incorporating the estimated FAR structure leads to substantially improved function estimates when compared to the initial results shown in the middle panel. These findings confirm the effectiveness of the proposed approach in refining the estimation of bivariate varying-coefficient functions by properly addressing the temporal dependence in the functional error process.

To further evaluate the performance of the proposed estimation procedure, we conduct simulations under varying sample sizes, specifically T=50,100 and n=50,100. The accuracy of the initial and improved estimators of $g_{m}\left(u,x_{m}\right)$ is assessed using the root mean squared error (RMSE), defined as

$$RMSE\left({\dot{g}}_{m}\right)=\frac{1}{T}\sum_{t=1}^{T}\{\frac{1}{n}\sum_{i=1}^{n}\left|\right|{\dot{g}}_{m}\left(u_{i},x_{t,m}\right)-g_{m}\left(u_{i},x_{t,m}\right){\left|\right|}_{2}^{2}{\}}^{\frac{1}{2}}.$$

where ${\dot{g}}_{m}$ denotes either the initial estimate ${\tilde{g}}_{m}$ or the improved estimate ${\hat{g}}_{m}$.

Table 1 presents the average root mean square errors (RMSEs) along with their standard deviations, calculated over 200 Monte Carlo replications for both the initial and improved estimators of $g_{m}\left(u,x_{m}\right)$. The results demonstrate a clear trend that the RMSEs decrease as both the number of time points *T* and the number of observations per curve *n* increase. More importantly, the improved estimators consistently outperform the initial estimators, yielding substantially lower RMSEs across all settings. This improvement is anticipated, as the initial estimates are obtained without adjusting for the auto-regressive error structure, which introduces bias and additional variability into the estimation process. In contrast, the improved estimates incorporate the estimated error component, leading to more accurate and reliable results.

To offer a deeper understanding of the relative performance between the two estimation strategies, we also compare their biases and standard deviations, taking the first setting in the simulation study as a representative example.

Table 2 presents the average bias and standard deviation of both the initial and improved estimators of $g_{m}\left(u,x_{m}\right)$. The results further confirm the superiority of the improved approach, indicating that across all combinations of sample size, both the bias and standard deviation of the improved estimators are markedly smaller than those of the initial estimators. Furthermore, both metrics exhibit a decreasing trend as the sample size increases, highlighting the consistency and efficiency of the improved estimation method. These findings provide strong empirical support for the theoretical result that the improved estimator possesses a smaller asymptotic variance–covariance matrix, thereby offering enhanced precision and robustness in practical applications.

### 5.2. Case 2

Case 2 is designed to evaluate the efficiency of identifying the auto-regressive order of the functional error process. The response densities are also generated from model (12), but now the error process follows a FAR(2) structure, with the second-order coefficient function specified as $\gamma_{2}\left(s,u\right)=\frac{1}{4}us^{2}$. All other simulation settings remain consistent with those in Case 1.

Table 3 reports the empirical power of the testing procedure used to determine the order of the FAR error process across various sample sizes and significance levels. The results clearly show that the test’s power increases as both the number of time points *T* and the number of observations per curve *n* grow. In particular, the power approaches one when *T* and *n* reach 100, especially when testing the null hypothesis of independent and identically distributed (i.i.d.) errors. This indicates that the test becomes highly reliable with larger sample sizes. While the power is somewhat lower when testing the null hypothesis of FAR(1) against FAR(2), this is expected due to the inherent difficulty in distinguishing between these closely related models. Additionally, the test maintains an appropriate size when assessing FAR(2) against higher-order alternatives, confirming its accuracy and practical feasibility for identifying the correct order of the functional error process. Overall, these findings demonstrate the robustness and effectiveness of the proposed testing algorithm in diverse settings.

To further explore the impact of correctly identifying the auto-regressive order *p* on estimation accuracy, Table 4 presents the average RMSEs of the bivariate varying-coefficient functions. The observed pattern closely parallels the results in Case 1, reinforcing the validity and reliability of the model’s identification and estimation procedures. This evidence highlights the critical role that the accurate determination of the auto-regressive order plays in improving estimation precision. The consistent RMSE patterns across different sample sizes and scenarios underline the model’s robustness in effectively accounting for the error structure, thus providing precise and reliable estimates of the bivariate varying-coefficient functions.

### 5.3. Case 3

To further examine the performance of proposed estimation approach and identification procedure under different scenarios, we consider the coefficient functions and innovation process which takes form as

$$\gamma_{1}\left(s,u\right)=0.2us,\;e_{t}\left(u\right)=0.2\eta_{t,1}\sin\left(\pi u\right)+\eta_{t,2}\sin\left(2\pi u\right),$$

with $\eta_{t,1}∼Gamma\left(3,2\right)$, $\eta_{t,2}∼t\left(5\right)$, and $\eta_{t,1}$ being independent of $\eta_{t,2}$ for u∈[0,1]. All other simulation settings remain consistent with those in Case 2.

Table 5 summarizes the power performance of the proposed testing approach under a setting where the innovation process $e_{t}\left(u\right)$ follows a non-Gaussian distribution with increased variability. The outcomes indicate that, similar to Case 2, the test remains capable of effectively identifying the correct order of the FAR process, even in the presence of more complex error structures. Although the overall power is somewhat increased relative to the non-Gaussian innovation process case, particularly in distinguishing closely related models such as FAR(1) versus FAR(2), the test still demonstrates satisfactory performance, especially as the sample size increases. Notably, when both the number of time points *T* and the number of observations per curve *n* are large (e.g., 100), the power approaches nearly unity, confirming that the test is still reliable under more challenging, non-ideal conditions.

Table 6 further examines how accurately identifying the auto-regressive order influences the estimation quality of the bivariate varying-coefficient functions. Despite the non-Gaussian error distribution and larger noise fluctuations leading to visibly higher RMSEs—both for the initial and refined estimates—consistent improvements are observed when the correct FAR order is utilized. These improvements closely mirror the trends seen in Case 2, reaffirming the stability and practical value of the estimation procedure. The results suggest that, although the estimation becomes inherently more difficult under heavy-tailed or heteroskedastic error conditions, the proposed methods remain applicable and beneficial in terms of both model identification and estimation refinement.

## 6. Real Data Analysis

In this section, we illustrate the feasibility and effectiveness of the proposed estimation procedure through the analysis of two real-world datasets. By applying our methodology to empirical data, we demonstrate its practical capability to capture the underlying patterns and dependencies present in complex data. This analysis not only serves to validate the performance of the estimation approach but also underscores its broad applicability across diverse domains. Moreover, it highlights the model’s flexibility and robustness in handling intricate, time-dependent, and non-Euclidean data structures, thus emphasizing its value as a versatile tool for real-world applications.

### 6.1. COVID-19 Data

On 11 March 2020, the World Health Organization (WHO) officially declared COVID-19, a contagious disease caused by the severe acute respiratory syndrome coronavirus 2 (SARS-CoV-2), a global pandemic. The rapid and widespread transmission of the virus presented unprecedented challenges to global public health, prompting countries worldwide to implement lockdowns and other measures aimed at controlling the spread of the disease. As of 15 August 2021, WHO reports indicated a staggering 221,885,822 confirmed cases and 4,583,539 deaths spanning nearly all countries, underscoring the extensive and profound impact of the pandemic. Given the scale of this crisis, it is crucial for international health organizations and research institutions to continuously monitor the evolving global trends of COVID-19. Such monitoring enables timely, accurate analysis that supports effective public health responses, informs medical treatment strategies, and guides prevention and control measures for future outbreaks. Understanding the epidemic’s dynamics through data-driven modeling is therefore essential for shaping informed policy decisions and improving health outcomes worldwide amid this ongoing global crisis.

To illustrate this point, we focus on the mortality rate as a key indicator for tracking the global trend of the COVID-19 pandemic. The mortality rate is defined as the ratio of the cumulative number of deaths each day to the total population of each country, serving as a critical measure of the disease’s lethality and spread. Importantly, the calculation of mortality rates inherently involves temporal dependence, as daily figures are based on previous days’ data. Consequently, the mortality rates, and thus the global epidemic trend, exhibit temporal autocorrelation.

The data on COVID-19-related deaths, essential to our analysis, are sourced from the publicly accessible Johns Hopkins University repository. This resource provides a dynamic tracking map offering comprehensive insights into global pandemic trends. The dataset, available at https://www.jhu.edu/ (accessed on 25 July 2021), covers the period from 22 January 2020 through 15 April 2021. Additionally, the most recent population data required to compute mortality rates for each country are obtained from the World Bank’s online platform, accessible at https://data.worldbank.org (accessed on 17 September 2021). These publicly available datasets form a valuable foundation for monitoring the pandemic’s progression and conducting rigorous statistical and epidemiological analyses to better understand the disease’s behavior across regions.

Because the timing of outbreaks varies between countries and regions, we standardize the time scale by defining day zero as the date when each country reached 100 cumulative confirmed COVID-19 cases. Our analysis considers daily cumulative death data from 189 countries over the subsequent 100-day period. At each time point *t*, we estimate the density function of the mortality rate, denoted as ${\hat{d}}_{t}\left(y\right)$, using data from these countries. Figure 1a displays the estimated densities of the global mortality rate (‰) across the 100 days, with data from up to 189 countries at each time point. Figure 1b presents an alternative view by showing the estimated densities on three selected days. From these visualizations, it is clear that the mortality rate densities remain well-defined throughout the observed period. Moreover, a temporal dependency among the distributions is clearly observable, suggesting the presence of an auto-regressive structure in the data, which supports the hypothesis of a functional auto-regressive (FAR) error process.

The main objective of this analysis, based on the COVID-19 data, is to identify the FAR process underlying the mortality rate and estimate its component functions. For the sake of simplicity, we begin by considering a special case where the covariate *z* is constant (set to 1), and *x* represents a scaled time variable, denoted as t/T. The model is specified as

$${\hat{f}}_{t}\left(u\right)=\Psi \left({\hat{d}}_{t}\right)\left(u\right)=g_{1}\left(u,x_{t,1}\right)+\varepsilon_{t}\left(u\right),\;1\leq t\leq 100,$$

where $\varepsilon_{t}\left(u\right)=\sum_{l=1}^{p}\int \gamma_{l}\left(u,s\right)\varepsilon_{t-l}\left(s\right)ds+e_{t}\left(u\right)$ and $x_{t,1}$ denotes the time scale t/T.

Using the initial spline estimate of $g_{1}\left(u,x_{1}\right)$, we apply a testing algorithm to determine the order *p* of the FAR process. Table 7 reports the corresponding *p*-values under different hypotheses. The results provide strong evidence of significant autocorrelation in the data, supporting the model of a first-order functional auto-regressive process, FAR(1). These findings empirically confirm the presence of temporal dependencies in the COVID-19 mortality rates, further justifying the application the use of a FAR error structure to effectively capture the evolving epidemic dynamics.

**Figure 4:**
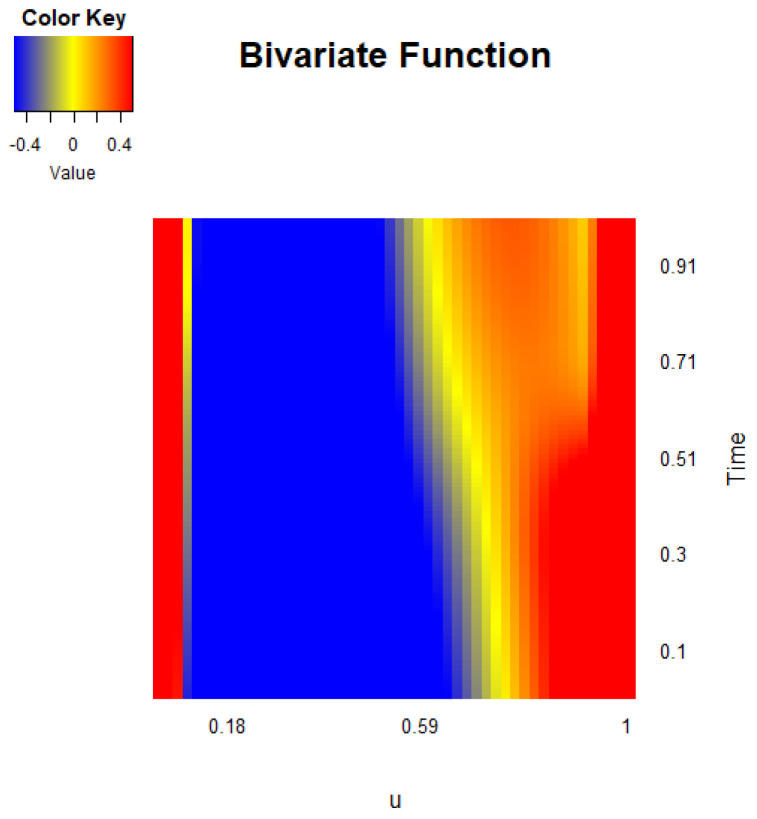
Heat map of bivariate varying-coefficient function $g_{1}\left(u,x_{1}\right)$ in the model based on the COVID-19 mortality rate (‰) data.

Figure 4 presents a heat map of the estimated bivariate function ${\hat{g}}_{1}\left(u,x_{1}\right)$, obtained after accounting for the functional error process and determining the auto-regressive order. The heat map reveals a relatively stable temporal pattern, where the function initially attains a minimum at lower values of *u*, gradually increases, and reaches a maximum at later time points. This pattern reflects the underlying dynamics of the COVID-19 mortality rate over successive days. The observed correlation between consecutive days further supports the notion that the global mortality rate exhibits substantial temporal dependence, consistent with the nature of the mortality measure derived from prior daily data.

To evaluate the uncertainty associated with these estimates, we conducted a residual-based bootstrap analysis. Specifically, we first fitted the model to obtain the estimated coefficient surface ${\hat{g}}_{1}\left(u,x_{1}\right)$ and the residual functions. Then, using the estimated functional auto-regressive operator from the FAR(1) error process, we recursively generated bootstrap residual samples by resampling the innovation functions with replacement. For each of the 500 bootstrap replications, new response functions were constructed by adding the bootstrap residuals to the fitted values based on ${\hat{g}}_{1}\left(u,x_{1}\right)$. The entire estimation procedure was repeated on each bootstrap dataset to obtain bootstrap replicates of the coefficient surface. The variability among these bootstrap replicates was then used to calculate point-wise standard errors and confidence intervals. The resulting standard errors were generally small across the domain, typically ranging between 0.04 and 0.07, indicating stable estimates throughout. The 95% confidence intervals for the bivariate varying-coefficient surface consistently excluded zero along the increasing temporal trend, confirming its statistical significance. Moreover, the bootstrap results showed that the identified pattern, a minimum at early *u* values followed by a gradual rise, was robust across replications, demonstrating that the observed dynamic is unlikely to be due to random noise. These findings underscore the reliability of the estimated surface and validate the importance of accounting for the temporal dependence captured by the FAR(1) process in modeling the functional response. This suggests that the observed structure is not an artifact of noise, but reflects a meaningful underlying dynamic, which reinforces the reliability of the visual patterns in the figure. Overall, these findings reinforce the necessity of incorporating a functional auto-regressive process to accurately model the temporal structure of the mortality rate. The clear and significant progression observed in the heat map further validates the model’s ability to capture the global evolution of the pandemic over time.

### 6.2. USA Income Data

Personal income statistics are essential for enabling governments to understand the interplay between national income, consumption, and saving. These statistics also serve as a valuable tool for assessing and comparing economic well-being across different regions or countries. In this study, we focus on the density time series of per capita personal income, defined as the total personal income of a region divided by its population. This metric offers a detailed perspective on the economic conditions within a region by capturing the distribution and evolution of income on a per-person basis over time. Analyzing such time series allows policymakers and researchers to gain insights into the long-term economic trends of a region, evaluate income disparities, and make more informed decisions regarding fiscal policies, social welfare programs, and strategies for economic development.

Income data for the United States are publicly publicly accessible through the official website of the United States Bureau of Economic Analysis (http://www.bea.gov/ accessed on 16 October 2021). We consider the quarterly per capita personal income of all 50 states in the USA spanning from the first quarter of 2010 through the fourth quarter of 2020, resulting in 44 time points, t=1,⋯,44. At each quarter *t*, we estimte the density function of per capita income, ${\hat{d}}_{t}\left(y\right)$, based on these 50 observations. Given that the quarterly personal income reflects broader national economic conditions, we incorporate two related covariates, ‘GDP’ (quarterly gross domestic product of the USA) and ‘Population’ (quarterly total population of the USA), both also available from the BEA (http://www.bea.gov/ accessed on 16 October 2021).

Traditionally, income curves are studied as panel data in economics, focusing on the relationship between consumers’ equilibrium points. As individual incomes fluctuate, the connections among these equilibrium points form trajectories that represent not only income growth but also increased consumer satisfaction. This perspective highlights the dynamic nature of income changes and their impact on well-being, offering valuable insights into consumer behavior over time.

In contrast, the income density curve, treated here as functional data, captures the distribution of income within a region or demographic group. It visually represents the shape and trends of income across different intervals, providing a more comprehensive view of the socio-economic environment. By examining income density curves, one can effectively observe income inequality within a population and identify key patterns of wealth distribution. Such curves are critical for economic research as they facilitate a deeper understanding of consumption behavior, socio-economic status, and the design of social policies.

Furthermore, income density curves are important tools for economic forecasting and analysis. By tracking changes in income distribution over time, economists can integrate insights about consumer preferences and consumption habits at different income levels. This approach enhances the ability to predict future economic conditions and shifts in consumption patterns, making income density curves indispensable for both microeconomic and macroeconomic analyses. Consequently, these curves play a crucial role in shaping policy decisions, economic planning, and our broader comprehension of economic well-being.

**Figure 5:**
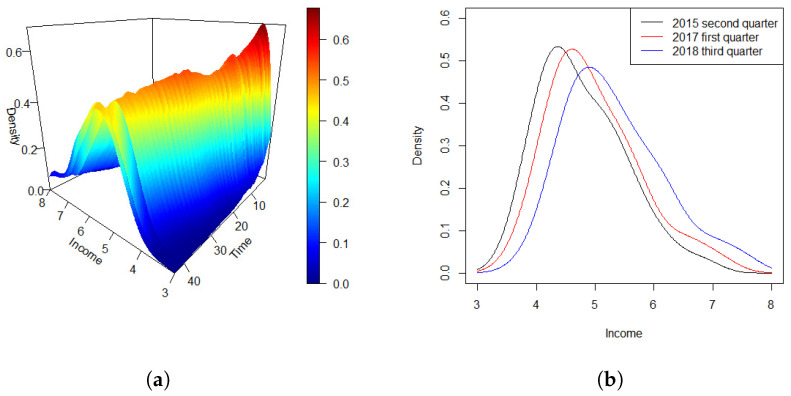
Densities of national quarterly personal income in the USA over 44 quarters. (**a**) Three-dimensional view of the density time series over the entire period; (**b**) density curves at three selected quarters.

Figure 5a depicts the density time series of quarterly personal income across the 44 quarters. The density curves indicate a consistent pattern in the distribution of per capita income across states over the past decade. Specifically, there are relatively few individuals in the high-income and upper-middle-income brackets, a moderate number in the middle-income category, and a larger share in the lower-middle-income range.

To illustrate the temporal changes in income distribution more clearly, Figure 5b shows density curves at three distinct time points: the second quarter of 2015, the first quarter of 2017, and the third quarter of 2018. The curves reveal a gradual shift towards higher income levels over time, alongside a corresponding decrease in the peak density. This trend aligns with broader economic and technological progress in recent years. As the economy develops, the proportion of individuals in lower income brackets steadily decreases, while the middle-to-high income groups grow. As a result, income distribution is becoming more balanced, with an increasing share of the population moving into middle- and higher-income categories. This pattern reflects general trends of economic growth and income redistribution over the period.

We model the income density curves using the following dynamic varying-coefficient auto-regressive functional regression (DVCA-FAR) model:

$${\hat{f}}_{t}\left(u\right)=\Psi \left({\hat{d}}_{t}\right)\left(u\right)=g_{0}\left(u,x_{t,0}\right)+z_{t,1}g_{1}\left(u,x_{t,1}\right)+z_{t,2}g_{2}\left(u,x_{t,2}\right)+\varepsilon_{t}\left(u\right),\;1\leq t\leq 44,$$

where $\varepsilon_{t}\left(u\right)=\sum_{l=1}^{p}\int \gamma_{l}\left(u,s\right)\varepsilon_{t-l}\left(s\right)ds+e_{t}\left(u\right)$. Here, $z_{t,1}$ denotes the quarterly gross domestic product of the USA, $z_{t,2}$ denotes the quarterly total population of the USA, and $x_{t,0},x_{t,1},x_{t,2}$ represent scaled time variables t/T.

Following the initial spline-based estimation, we apply a testing procedure to determine the appropriate order *p* of the functional auto-regressive error process. The *p*-values in Table 8 indicate that an FAR(2) model best captures the autocorrelation structure in the error terms. This finding suggests that a second-order functional auto-regressive process effectively accounts for the dynamic dependencies within the income data.

**Figure 6:**
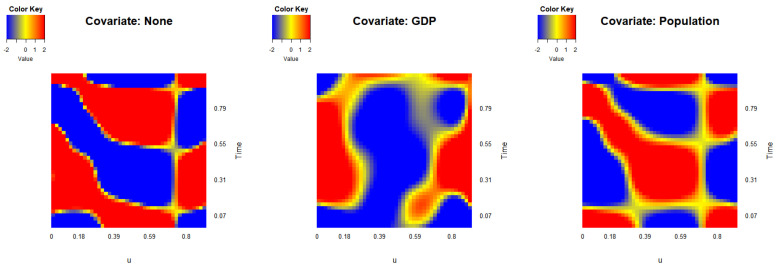
Heat maps of bivariate varying-coefficient functions $g_{m}\left(u,x_{m}\right),m=0,1,2,$ based on the USA income data.

Using the three-step estimation procedure, we estimate the bivariate varying-coefficient functions. Figure 6 presents heat maps of the three estimated functions, where $g_{0}\left(u,x_{0}\right)$ represents the baseline effect over time, $g_{1}\left(u,x_{1}\right)$ captures the influence of GDP, and $g_{2}\left(u,x_{2}\right)$ reflects the impact of population on data respectively. The heat map of $g_{0}$ shows an alternating pattern over time between high and low values, indicating that individuals at both high and low per capita income levels experience similar effects, whereas those in the middle-income range tend to display the opposite trend. The function $g_{1}$, related to GDP, exhibits a consistent pattern across income levels *u* that changes over time, with an initial peak followed by a dip and a subsequent rise toward the end of the period. Conversely, the effect of population, as shown in $g_{2}$, generally opposes the baseline pattern and remains relatively stable across both high- and low-income groups.

To evaluate the uncertainty associated with these estimated varying-coefficient surfaces, we conducted a residual-based bootstrap analysis analogous to that described previously. Specifically, we used the estimated FAR(2) operator and innovation residuals to generate 500 bootstrap samples, each time reconstructing the response functions and refitting the entire model. Point-wise standard errors and confidence intervals for all $g_{j}\left(u,x_{j}\right)$ derived from these bootstrap replications indicate that the main features of the estimated coefficient functions are statistically significant and robust throughout the domain. Point-wise standard errors and confidence intervals for all estimated coefficient functions $g_{j}\left(u,x_{j}\right)$ obtained from the 500 bootstrap replications show that the estimation uncertainty is generally low across most of the domain. For example, the standard errors of $g_{0}\left(u,x_{0}\right)$ remain below 0.25 in regions corresponding to the high- and low-income groups, supporting the stability of the observed alternating pattern over time. Similarly, $g_{1}\left(u,x_{1}\right)$, representing the GDP effect, exhibits standard errors typically under 0.38 near the temporal peaks and troughs, confirming that the initial peak, mid-period dip, and late-period rise are statistically significant features rather than random fluctuations. The function $g_{2}\left(u,x_{2}\right)$, reflecting population impact, has slightly larger uncertainty in the mid-income range but remains stable and significant across the majority of the income spectrum. Overall, the 95% bootstrap confidence intervals exclude zero in these key regions, reinforcing the robustness and reliability of the identified macroeconomic influences on quarterly personal income distributions. Together, these findings highlight a significant dependence of quarterly personal income distributions in the United States on prior values, with the dynamics closely linked to key macroeconomic factors such as GDP and demographic changes represented by population growth.

## 7. Conclusions

Sequentially collected data often exhibit autocorrelation, which must be properly addressed to ensure accurate statistical modeling. At the same time, the analysis of non-Euclidean data structures, such as probability density functions, has gained increasing attention in modern statistical research. To address these challenges, we propose a varying-coefficient additive model with density-valued responses, incorporating a functional auto-regressive (FAR) error process to capture temporal dependence. Given the intrinsic nonlinearity and geometric constraints of density functions, we begin by applying the transformation method proposed by Petersen and Müller [6] to map the density functions into a linear Hilbert space, enabling the use of conventional regression techniques. We then develop a three-step estimation procedure for the varying-coefficient components. In the first step, we employ B-spline series approximations to obtain preliminary estimates of the bivariate varying-coefficient functions, initially ignoring the functional error structure. In the second step, we determine the order of the FAR process using the test statistic introduced by Kokoszka and Reimherr [16], based on the residuals obtained from the first step. In the final step, we account for the FAR error process and construct refined estimators for the varying-coefficient functions by removing the estimated auto-correlated components and reapplying the B-spline estimation. We provide theoretical justification for the proposed procedure by establishing convergence rates and asymptotic properties for both the initial and refined estimators. The effectiveness of the proposed method is further demonstrated through comprehensive simulation studies and applications to two real-world datasets. The results underscore the importance of addressing temporal dependence in density-valued data and validate the accuracy and efficiency of our approach.

This work opens several avenues for future research. While our model establishes the relationship between density function responses and scalar predictors using a varying-coefficient additive framework, the growing prevalence of complex, high-dimensional data calls for further methodological extensions. In particular, although a FAR structure is assumed for temporal dependence, consistent estimation may still be possible under simpler or approximate structures, similar to working correlations in GEE. Exploring such alternatives could offer more flexible and efficient modeling in future work. While the estimation method proposed in this article combines well-established techniques, its tailored integration within the context of density time series with functional auto-regressive errors addresses unique challenges in this setting. Nevertheless, developing more novel and efficient estimation approaches remains an important direction for future research, with potential to further improve accuracy and computational performance. Furthermore, although the least squares-based estimation method used in this paper is not optimal in the classical sense due to the presence of temporally dependent functional errors, it remains a theoretically justified and practically effective approach in our setting. The estimator achieves consistency and asymptotic normality, and is tailored to the model’s structural complexity. Developing more efficient alternatives, such as methods that explicitly incorporate the error dependence structure, represents a promising direction for future research. Our work adapts classical theoretical tools to this complex setting, but there exist additional methods and frameworks that could further strengthen the theoretical properties. Developing and applying these tools offers valuable opportunities for future research. Additionally, a limitation of the current approach is that it models the distribution of responses pooled across units at each time point, thus not capturing individual unit trajectories over time. This means that development within specific countries or states cannot be directly traced. Extending the model to include unit-specific effects or hierarchical structures, such as functional mixed-effects models, would allow for tracking of within-unit temporal dynamics while accounting for autocorrelation. Future studies will aim to develop such extensions and explore their theoretical and empirical properties in greater depth.

## Figures and Tables

**Table 1:** Average RMSEs of both initial and improved estimators of bivariate varying-coefficient additive functions $g_{m}\left(u,x_{m}\right)$.

Average RMSEs of Bivariate Varying-Coefficient Additive Functions					
Sample Size		$g_{1}\left(u,x_{1}\right)$		$g_{2}\left(u,x_{2}\right)$	
T	n	Initial	Improved	Initial	Improved
50	50	0.2247	0.1848	0.2139	0.1785
	100	0.1759	0.1325	0.1844	0.1521
100	50	0.1826	0.1471	0.1732	0.1354
	100	0.1431	0.1164	0.1319	0.1057

**Table 2:** Average Standard Deviation (SD) and Bias of both initial and improved estimators of bivariate varying-coefficient additive functions $g_{m}\left(u,x_{m}\right)$.

Average SD and Bias of Bivariate Varying-Coefficient Additive Functions									
Sample Size		$g_{1}\left(u,x_{1}\right)$				$g_{2}\left(u,x_{2}\right)$			
		Initial		Improved		Initial		Improved	
T	n	SD	Bias	SD	Bias	SD	Bias	SD	Bias
50	50	0.205	0.147	0.168	0.104	0.219	0.137	0.183	0.117
	100	0.179	0.122	0.142	0.093	0.196	0.128	0.164	0.095
100	50	0.174	0.136	0.151	0.082	0.187	0.131	0.158	0.086
	100	0.133	0.099	0.112	0.057	0.153	0.111	0.129	0.061

**Table 3:** Empirical power of testing algorithm to determine the order of FAR error process under different significance levels.

Null Hypothesis		p=0		p≤1		p≤2	
Alternative Hypothesis		p≥1		p≥2		p≥3	
Sample Size		Significance Level		Significance Level		Significance Level	
T	n	0.05	0.1	0.05	0.1	0.05	0.1
50	50	0.893	0.962	0.787	0.846	0.082	0.134
	100	0.931	0.985	0.824	0.893	0.073	0.125
100	50	0.942	0.972	0.821	0.881	0.071	0.121
	100	0.985	1.000	0.889	0.935	0.064	0.113

**Table 4:** Average RMSEs of both initial and improved estimators of bivariate varying-coefficient additive functions $g_{m}\left(u,x_{m}\right)$.

Average RMSEs of Bivariate Varying-Coefficient Additive Functions					
Sample Size		$g_{1}\left(u,x_{1}\right)$		$g_{2}\left(u,x_{2}\right)$	
T	n	Initial	Improved	Initial	Improved
50	50	0.2739	0.2438	0.2691	0.2235
	100	0.2264	0.1852	0.2157	0.1809
100	50	0.2136	0.1817	0.2232	0.1761
	100	0.1729	0.1263	0.1816	0.1224

**Table 5:** Empirical power of testing algorithm to determine the order of FAR error process under different significance levels.

Null Hypothesis		p=0		p≤1		p≤2	
Alternative Hypothesis		p≥1		p≥2		p≥3	
Sample Size		Significance Level		Significance Level		Significance Level	
T	n	0.05	0.1	0.05	0.1	0.05	0.1
50	50	0.832	0.891	0.724	0.795	0.154	0.197
	100	0.876	0.932	0.776	0.843	0.136	0.162
100	50	0.884	0.937	0.792	0.838	0.131	0.159
	100	0.923	0.951	0.854	0.893	0.089	0.127

**Table 6:** Average RMSEs of both initial and improved estimators of bivariate varying-coefficient additive functions $g_{m}\left(u,x_{m}\right)$.

Average RMSEs of Bivariate Varying-Coefficient Additive Functions					
Sample Size		$g_{1}\left(u,x_{1}\right)$		$g_{2}\left(u,x_{2}\right)$	
T	n	Initial	Improved	Initial	Improved
50	50	0.5379	0.4906	0.5582	0.5072
	100	0.4631	0.4125	0.4824	0.4436
100	50	0.4582	0.4207	0.4871	0.4320
	100	0.3965	0.3518	0.4241	0.3736

**Table 7:** *p*-values from the testing algorithm applied to identify the order of functional error process based on the mortality rate data of COVID-19.

Null Hypothesis	p=0	p≤1
Alternative Hypothesis	p≥1	p≥2
*p*-value	0.000	0.194

**Table 8:** *p*-values from the testing algorithm for determining the order of the functional error process based on the USA income data.

Null Hypothesis	p=0	p≤1	p≤2
Alternative Hypothesis	p≥1	p≥2	p≥3
*p*-value	0.000	0.000	0.436

## Data Availability

The original datasets employed in this study are publicly accessible from the official website of Johns Hopkins University at https://www.jhu.edu/ (accessed on 25 July 2021), the World Bank’s online platform at https://data.worldbank.org/ (accessed on 17 September 2021), and the United States Bureau of Economic Analysis at http://www.bea.gov/ (accessed on 16 October 2021).

## Supplementary Materials

The following supporting information can be downloaded at https://www.mdpi.com/article/10.3390/e27080882/s1. The supplementary materials provide a detailed proof of the theoretical results. Refs. [18,19] are cited in the supplementary materials.

## References

[1] Kokoszka P., Miao H., Petersen A., Shang H.L. (2019). Forecasting of density functions with an application to cross-sectional and intraday returns. Int. J. Forecast..

[2] Sen R., Ma C. (2015). Forecasting density function: Application in finance. J. Math. Financ..

[3] Petersen A., Müller H. (2019). Fréchet regression for random objects with Euclidean predictors. Ann. Stat..

[4] Petersen A., Chen C., Müller H. (2019). Quantifying and visualizing intraregional connectivity in resting-state functional magnetic resonance imaging with correlation densities. Brain Connect..

[5] Saha A., Banerjee S., Kurtek S., Narang S., Lee J., Rao G., Martinez J., Bharath K., Rao A., Baladandayuthapani V. (2016). DEMARCATE: Density-based magnetic resonance image clustering for assessing tumor heterogeneity in cancer. NeuroImage Clin..

[6] Petersen A., Müller H. (2016). Functional data analysis for density functions by transformation to a Hilbert space. Ann. Stat..

[7] Han K., Müller H., Park B. (2020). Additive functional regression for densities as responses. J. Am. Stat. Assoc..

[8] Talská R., Menafoglio A., Machalová J., Hron K., Fiserová E. (2018). Compositional regression with functional response. Comput. Stat. Data Anal..

[9] Chen Y., Lin Z., Müller H. (2023). Wasserstein regression. J. Am. Stat. Assoc..

[10] Zhang C., Kokoszka P., Petersen A. (2022). Wasserstein autoregressive models for density time series. J. Time Ser. Anal..

[11] Berhoune K., Bensmain N. (2018). Sieves estimator of functional autoregressive process. Stat. Probab. Lett..

[12] Chen Y., Chua W.S., Hardle W. (2019). Forecasting limit order book liquidity supply-demand curves with functional autoregressive dynamics. Quant. Financ..

[13] Chen Y., Li B. (2017). An adaptive functional autoregressive forecast model to predict electricity price curves. J. Bus. Econ. Stat..

[14] Daniel R., David S., David R. (2019). Functional autoregression for sparsely sampled data. J. Bus. Econ. Stat..

[15] Bosq D. (2000). Linear Processes in Function Spaces: Theory and Applications.

[16] Kokoszka P., Reimherr M. (2013). Determining the order of the functional autoregressive model. J. Time Ser. Anal..

[17] Xu X., Chen Y., Zhang G., Koch T. (2022). Modeling functional time series and mixed-type predictors with partially functional autoregressions. J. Bus. Econ. Stat..

[18] Stone C. (1994). The use of polynomial splines and their tensor products in multivariate function estimation. Ann. Stat..

[19] DeVore R., Lorentz G. (1993). Constructive Approximation.

