# Supplementary material for: Varying-Coefficient Additive Models with Density Responses and Functional Auto-Regressive Error Process
Source: Entropy (Basel). 2025 Aug 20;27(8):882. doi: 10.3390/e27080882 (PMC12385778; doi:10.3390/e27080882)
Supplement: Supplementary file 1 [file entropy-27-00882-s001.zip › entropy-3781714-Supplement.pdf]

entropy

MDPI

Article

Supplementary Material - Varying-Coefficient Additive Models with Density Responses and Functional Auto-Regressive Error Process

Zixuan Han [1], Tao Li [2,*], Jinhong You [2] and Narayanaswamy Balakrishnan [3]

1 Division of Public Health Sciences, Fred Hutchinson Cancer Center, Seattle, WA 98109, USA
2 School of Statistics and Data Science, Shanghai University of Finance and Economics, Shanghai 200433, China
3 Department of Mathematics and Statistics, McMaster University, Hamilton, Ontario L8S 4L8, Canada
* Correspondence: li.tao@mail.shufe.edu.cn

In this supplementary material, we provide detailed proofs of the theoretical results established in the main text.

Proof of Theorem 1. As detailed in the main article, the proposed varying-coefficient additive model with a functional error process can be expressed as

$$f_t(u) = \sum_{m=1}^{k} z_{t,m} g_m(u, x_{t,m}) + \varepsilon_t(u), \quad 0 \le u \le 1.$$

Denote the vector of basis functions $\boldsymbol{b}(u, x_m) = (b_{1,1,m}(u, x_m), \cdots, b_{N_0,N_m,m}(u, x_m))^\tau$, and corresponding coefficient vectors $\boldsymbol{\lambda_m} = (\lambda_{1,1,m}, \cdots, \lambda_{N_0,N_m,m})^\tau$, $\tilde{\boldsymbol{\lambda}}_{\boldsymbol{m}} = (\tilde{\lambda}_{1,1,m}, \cdots, \tilde{\lambda}_{N_0,N_m,m})^\tau$ for each $m = 1, \cdots, k$. Further, we set $\boldsymbol{\lambda} = (\boldsymbol{\lambda_1}, \cdots, \boldsymbol{\lambda_k})^\tau$, and $\tilde{\boldsymbol{\lambda}} = (\tilde{\boldsymbol{\lambda}}_{\boldsymbol{1}}, \cdots, \tilde{\boldsymbol{\lambda}}_{\boldsymbol{k}})^\tau$.

Using the spline approximation method, the bivariate function $g_m(u, x_m)$ can be represented in the matrix form as

$$g_m(u, x_m) \approx \sum_{r=1}^{N_0} \sum_{j=1}^{N_m} \lambda_{r,j,m} b_{r,j,m}(u, x_m) = \boldsymbol{b}^\tau(u, x_m) \boldsymbol{\lambda_m}.$$

Ignoring the *FAR* error term, the initial estimator of bivariate varying-coefficient functions $g_m(u, x_m)$ is given by

$$\tilde{g}_m(u, x_m) = \sum_{r=1}^{N_0} \sum_{j=1}^{N_m} \tilde{\lambda}_{r,j,m} b_{r,j,m}(u, x_m) = \boldsymbol{b}^\tau(u, x_m) \tilde{\boldsymbol{\lambda}}_{\boldsymbol{m}}, \quad 1 \le m \le k,$$

where $\tilde{\boldsymbol{\lambda}} = (\tilde{\lambda}_{1,1,1}, \cdots, \tilde{\lambda}_{N_0,N_k,k})^\tau$ is a $(N_0 \sum_{m=1}^{k} N_m)$-dimensional vector obtained by solving

$$\tilde{\boldsymbol{\lambda}} = \arg\min_{\boldsymbol{\lambda}} \sum_{t=1}^{T} \sum_{i=1}^{n} \left[f_t(u_i) - \sum_{m=1}^{k} z_{t,m} \sum_{r=1}^{N_0} \sum_{j=1}^{N_m} \lambda_{r,j,m} b_{r,j,m}(u_i, x_{t,m}) \right]^2.$$

We first introduce additional notation for convenience. Denote $\boldsymbol{B} = (\boldsymbol{B_1}, \cdots, \boldsymbol{B_k})$, $\boldsymbol{B_m} = (\boldsymbol{Bz_{1,m}}^\tau, \cdots, \boldsymbol{Bz_{T,m}}^\tau)^\tau$, where $\boldsymbol{Bz_{t,m}} = (\boldsymbol{b_z}(u_1, x_{t,m}), \cdots, \boldsymbol{b_z}(u_n, x_{t,m}))^\tau$, and $\boldsymbol{b_z}(u, x_{t,m}) = z_{t,m} \boldsymbol{b}(u, x_{t,m})$. Further, define $\boldsymbol{f_t} = (f_t(u_1), \cdots, f_t(u_n))^\tau$, $\boldsymbol{f} = (\boldsymbol{f_1}^\tau, \cdots, \boldsymbol{f_T}^\tau)^\tau$, and $\boldsymbol{\varepsilon_t} = (\varepsilon_t(u_1), \cdots, \varepsilon_t(u_n))^\tau$, $\boldsymbol{\varepsilon} = (\boldsymbol{\varepsilon_1}^\tau, \cdots, \boldsymbol{\varepsilon_T}^\tau)^\tau$.

Let $\hat{f}_t(u) = \Psi(\hat{d}_t)(u)$ denote the estimator of $f_t(u)$ based on observations $\{Y_t\}$, and define $\hat{\boldsymbol{f}} = (\hat{\boldsymbol{f}}_{\boldsymbol{1}}^\tau, \cdots, \hat{\boldsymbol{f}}_{\boldsymbol{T}}^\tau)^\tau$, where $\hat{\boldsymbol{f}}_{\boldsymbol{t}} = (\hat{f}_t(u_1), \cdots, \hat{f}_t(u_n))^\tau$. It follows that $\hat{\boldsymbol{f}} = \boldsymbol{f} + \boldsymbol{\varepsilon_f}$, where $\boldsymbol{\varepsilon_f} = (\boldsymbol{\varepsilon_{f_1}}^\tau, \cdots, \boldsymbol{\varepsilon_{f_T}}^\tau)^\tau$, with $\boldsymbol{\varepsilon_{f_t}} = \Psi(\hat{d}_t) - \Psi(d_t)$ representing the error induced by

check for updates

Received: 11 July 2025
Revised: 15 August 2025
Accepted: 19 August 2025
Published: 20 August 2025

Citation: Han, Z.; Li, T.; You, J.; Balakrishnan, N. Supplementary Material - Varying-Coefficient Additive Models with Density Responses and Functional Auto-Regressive Error Process. *Entropy* **2025**, *27*, 882. https://doi.org/10.3390/e27080882

Copyright: © 2025 by the authors. Licensee MDPI, Basel, Switzerland. This article is an open access article distributed under the terms and conditions of the Creative Commons Attribution (CC BY) license (https://creativecommons.org/licenses/by/4.0/).

the LQD transformation of the density estimation $\hat{d}_t$. For each d_t, we assume the error arising from the transformation and kernel smoothing are identically and independent distributed. The estimator or the coefficient vector $\boldsymbol{\lambda}$ is given by $\tilde{\boldsymbol{\lambda}} = (\boldsymbol{B}^\tau \boldsymbol{B})^{-1}\boldsymbol{B}^\tau \hat{\boldsymbol{f}}$.

For simplicity, define $g_z(u, \boldsymbol{x_t}) = \sum_{m=1}^{k} z_{t,m} g_m(u, x_{t,m})$, $\boldsymbol{g_t} = (g_z(u_1, \boldsymbol{x_t}), \cdots, g_z(u_n, \boldsymbol{x_t}))^\tau$, and $\boldsymbol{g} = (\boldsymbol{g_1}^\tau, \cdots, \boldsymbol{g_T}^\tau)^\tau$. Let $\boldsymbol{A_m} = (\boldsymbol{0}, \cdots, \boldsymbol{I}, \cdots, \boldsymbol{0})$ denote the block selection matrix of dimension $N_0 N_m \times N_0 \sum_{i=1}^{k} N_i$ block matrix, structured with each block be an $N_0 N_m \times N_0 N_i$ matrix, $i = 1, \cdots, k$, such that the m-th block is an identity matrix and all other blocks are zero matrices.

To establish the consistency of the estimator $\tilde{g}_m(u, x_m)$, we first decompose the estimation error $\tilde{g}_m(u, x_m) - g_m(u, x_m)$ into three parts, which is as follows.

$$\begin{aligned}
&\tilde{g}_m(u, x_m) - g_m(u, x_m) \\
=&\boldsymbol{b}^\tau(u, x_m)\boldsymbol{A_m}(\boldsymbol{B}^\tau\boldsymbol{B})^{-1}\boldsymbol{B}^\tau\hat{\boldsymbol{f}} - g_m(u, x_m) \\
=&\boldsymbol{b}^\tau(u, x_m)\boldsymbol{A_m}(\boldsymbol{B}^\tau\boldsymbol{B})^{-1}\boldsymbol{B}^\tau\boldsymbol{f} + \boldsymbol{b}^\tau(u, x_m)\boldsymbol{A_m}(\boldsymbol{B}^\tau\boldsymbol{B})^{-1}\boldsymbol{B}^\tau\boldsymbol{\varepsilon_f} - g_m(u, x_m) \\
=&\boldsymbol{b}^\tau(u, x_m)\boldsymbol{A_m}(\boldsymbol{B}^\tau\boldsymbol{B})^{-1}\boldsymbol{B}^\tau\boldsymbol{g} - g_m(u, x_m) \\
&+ \boldsymbol{b}^\tau(u, x_m)\boldsymbol{A_m}(\boldsymbol{B}^\tau\boldsymbol{B})^{-1}\boldsymbol{B}^\tau\boldsymbol{\varepsilon} + \boldsymbol{b}^\tau(u, x_m)\boldsymbol{A_m}(\boldsymbol{B}^\tau\boldsymbol{B})^{-1}\boldsymbol{B}^\tau\boldsymbol{\varepsilon_f} \\
=&g^B(u, x_m) + g^V(u, x_m) + g^e(u, x_m),
\end{aligned}$$

where

$$\begin{aligned}
g^B(u, x_m) &= \boldsymbol{b}^\tau(u, x_m)\boldsymbol{A_m}(\boldsymbol{B}^\tau\boldsymbol{B})^{-1}\boldsymbol{B}^\tau\boldsymbol{g} - g_m(u, x_m), \\
g^V(u, x_m) &= \boldsymbol{b}^\tau(u, x_m)\boldsymbol{A_m}(\boldsymbol{B}^\tau\boldsymbol{B})^{-1}\boldsymbol{B}^\tau\boldsymbol{\varepsilon}, \\
g^e(u, x_m) &= \boldsymbol{b}^\tau(u, x_m)\boldsymbol{A_m}(\boldsymbol{B}^\tau\boldsymbol{B})^{-1}\boldsymbol{B}^\tau\boldsymbol{\varepsilon_f}.
\end{aligned}$$

Considering the bias term $g^B(u, x_m)$, we have

$$\begin{aligned}
&g^B(u, x_m) \\
=&\boldsymbol{b}^\tau(u, x_m)\boldsymbol{A_m}(\boldsymbol{B}^\tau\boldsymbol{B})^{-1}\boldsymbol{B}^\tau\boldsymbol{g} - g_m(u, x_m) \\
=&\boldsymbol{b}^\tau(u, x_m)\boldsymbol{A_m}\left[(\boldsymbol{B}^\tau\boldsymbol{B})^{-1}\boldsymbol{B}^\tau\boldsymbol{g} - \boldsymbol{\lambda}\right] + \boldsymbol{b}^\tau(u, x_m)\boldsymbol{A_m}\boldsymbol{\lambda} - g_m(u, x_m) \\
=&\boldsymbol{b}^\tau(u, x_m)\boldsymbol{A_m}(\boldsymbol{B}^\tau\boldsymbol{B})^{-1}\boldsymbol{B}^\tau[\boldsymbol{g} - \boldsymbol{B}\boldsymbol{\lambda}] + \left[\boldsymbol{b}^\tau(u, x_m)\boldsymbol{\lambda_m} - g_m(u, x_m)\right] \\
=&\boldsymbol{b}^\tau(u, x_m)\boldsymbol{A_m}(\frac{1}{nT}\boldsymbol{B}^\tau\boldsymbol{B})^{-1}\boldsymbol{B}^\tau\left[\frac{1}{nT}(\boldsymbol{g} - \boldsymbol{B}\boldsymbol{\lambda})\right] + \left[\boldsymbol{b}^\tau(u, x_m)\boldsymbol{\lambda_m} - g_m(u, x_m)\right].
\end{aligned}$$

According to [1], the traditional bivariate spline estimator achieves a convergence rate of order $O_p(N_m^{-2})$. In other words, there exists a constant C_0, such that

$$\sup_{u, x_m} |g_m(u, x_m) - \boldsymbol{b}(u, x_m)^\tau\boldsymbol{\lambda_m}| \leq C_0 N_m^{-2}.$$

For notation simplicity, we assume that there exists a constant N_1 such that $N_0 = N_m = N_1$, for all $1 \leq m \leq k$. It then follows that

$$\sup_{u,x} \frac{1}{nT}|\boldsymbol{g} - \boldsymbol{B}\boldsymbol{\lambda}| \leq C_1 N_1^{-2},$$

for some constant $C_1 > 0$. Combining this with the known result $||(\frac{1}{nT}\boldsymbol{B}^{\tau}\boldsymbol{B})^{-1}|| = O_p(N_1^2)$, which can be derived from [2], therefore, we can conclude that

$$\sup_{u,x_m \in [0,1]} |g^B(u, x_m)| = O_p(N_1^{-2}).$$

For the variance term, recall that $g^V(u, x_m) = \boldsymbol{b}^{\tau}(u, x_m)\boldsymbol{A_m}(\boldsymbol{B}^{\tau}\boldsymbol{B})^{-1}\boldsymbol{B}^{\tau}\boldsymbol{\varepsilon}$, where the error process $\boldsymbol{\varepsilon}$ follows a functional auto-regressive process, defined by

$$\varepsilon_t(u) = \sum_{l=1}^{p} \int \gamma_l(s, u)\varepsilon_{t-l}(s)ds + e_t(u),$$

and $\{\boldsymbol{e_t}\}_{t=1}^{T}$ is an i.i.d. innovation process satisfying $E(e_t(u)|\boldsymbol{x_t}, \boldsymbol{z_t}) = 0$. We further assume the largest eigenvalues of the covariance operator $\Sigma_{\varepsilon}(u)$, λ_{max}, is finite. Therefore, the expectation of $E(g^V(u, x_m))$ is thus

$$\begin{aligned} E(g^V(u, x_m)) &= E[E(g^V(u, x_m)|\boldsymbol{x}, \boldsymbol{z})] = E[E(\boldsymbol{b}^{\tau}(u, x_m)\boldsymbol{A_m}(\boldsymbol{B}^{\tau}\boldsymbol{B})^{-1}\boldsymbol{B}^{\tau}\boldsymbol{\varepsilon}|\boldsymbol{x}, \boldsymbol{z})] \\ &= E[\boldsymbol{b}^{\tau}(u, x_m)\boldsymbol{A_m}(\boldsymbol{B}^{\tau}\boldsymbol{B})^{-1}\boldsymbol{B}^{\tau}E(\boldsymbol{\varepsilon}|\boldsymbol{x}, \boldsymbol{z})] = 0. \end{aligned}$$

Next, we consider its second moment:

$$\begin{aligned} &E(g^V(u, x_m))^2 = E[E(g^V(u, x_m)^2|\boldsymbol{x}, \boldsymbol{z})] \\ =&E\left[E\left((\boldsymbol{b}^{\tau}(u, x_m)\boldsymbol{A_m}(\boldsymbol{B}^{\tau}\boldsymbol{B})^{-1}\boldsymbol{B}^{\tau}\boldsymbol{\varepsilon})^2|\boldsymbol{x}, \boldsymbol{z}\right)\right] \\ =&E\left[E\left(\boldsymbol{b}^{\tau}(u, x_m)\boldsymbol{A_m}(\boldsymbol{B}^{\tau}\boldsymbol{B})^{-1}\boldsymbol{B}^{\tau}\boldsymbol{\varepsilon}\boldsymbol{\varepsilon}^{\boldsymbol{\tau}}\boldsymbol{B}(\boldsymbol{B}^{\tau}\boldsymbol{B})^{-1}\boldsymbol{A_m}^{\tau}\boldsymbol{b}(u, x_m)|\boldsymbol{x}, \boldsymbol{z}\right)\right] \\ =&E\left[\boldsymbol{b}^{\tau}(u, x_m)\boldsymbol{A_m}(\boldsymbol{B}^{\tau}\boldsymbol{B})^{-1}\boldsymbol{B}^{\tau}E(\boldsymbol{\varepsilon}\boldsymbol{\varepsilon}^{\boldsymbol{\tau}}|\boldsymbol{x}, \boldsymbol{z})\boldsymbol{B}(\boldsymbol{B}^{\tau}\boldsymbol{B})^{-1}\boldsymbol{A_m}^{\tau}\boldsymbol{b}(u, x_m)\right] \\ =&\boldsymbol{b}^{\tau}(u, x_m)\boldsymbol{A_m}(\boldsymbol{B}^{\tau}\boldsymbol{B})^{-1}\boldsymbol{B}^{\tau}\boldsymbol{\Sigma_{\varepsilon}}\boldsymbol{B}(\boldsymbol{B}^{\tau}\boldsymbol{B})^{-1}\boldsymbol{A_m}^{\tau}\boldsymbol{b}(u, x_m). \end{aligned}$$

By bounding the covariance operator using Σ_{ε} and recognizing that $||\Sigma_{\varepsilon}(u)|| \leq C\lambda_{max}$, we obtain

$$E(g^V(u, x_m))^2 \leq \frac{C\lambda_{max}}{nT}\boldsymbol{b}^{\tau}(u, x_m)\boldsymbol{A_m}(\frac{1}{nT}\boldsymbol{B}^{\tau}\boldsymbol{B})^{-1}\boldsymbol{A_m}^{\tau}\boldsymbol{b}(u, x_m).$$

Finally, applying the operator norm

$$E(g^V(u, x_m))^2 \leq \frac{C\lambda_{max}}{nT}\boldsymbol{b}(u, x_m)\boldsymbol{b}^{\tau}(u, x_m)\left|\left|(\frac{1}{nT}\boldsymbol{B}^{\tau}\boldsymbol{B})^{-1}\right|\right|.$$

Given the spline basis dimension N_1, and under standard conditions [2], we have

$$\sup_{u,x_m \in [0,1]} |g^V(u, x_m)| = O_p(\sqrt{N_1/nT}) = O_p\left(N_1^{1/2}/\sqrt{nT}\right).$$

For the transformation-based error term, recall that

$$g^e(u, x_m) = \boldsymbol{b}^{\tau}(u, x_m)\boldsymbol{A_m}(\boldsymbol{B}^{\tau}\boldsymbol{B})^{-1}\boldsymbol{B}^{\tau}\boldsymbol{\varepsilon_f},$$

where $f_t(u) = \Psi(d_t)(u)$ and the estimation obtained from observations is $\hat{f}_t(u) = \Psi(\hat{d}_t(u))$. According to [3], the density estimation satisfies

$$\sup_{d_t \in \mathcal{F}} |\hat{d}_t - d_t| = O_p(h + (nh)^{-1/2}).$$

Due to the smoothness and Lipschitz continuity of the log-quantile density (LQD) transformation, this error propagates as:

$$\sup_{d_t} |\Psi(\hat{d}_t) - \Psi(d_t)| = O_p(h + (nh)^{-1/2}).$$

Threfore, the error term satisfies:

$$\sup_{u,x_m \in [0,1]} |g^e(u, x_m)| = O_p(h + (nh)^{-\frac{1}{2}}).$$

Combining the bias, variance, and transformation error terms, assuming $h \sim n^{-1/3}$, $N_0, N_m \sim (nT)^{1/6} \log nT$, thus $N_1 \sim (nT)^{1/6} \log nT$, as $n, T \to \infty$, we have

$$\begin{aligned}
&\sup_{u,x_m \in [0,1]} |\tilde{g}_m(u, x_m) - g_m(u, x_m)| \\
\leq &\sup_{u,x_m \in [0,1]} |g^B(u, x_m)| + \sup_{u,x_m \in [0,1]} |g^V(u, x_m)| + \sup_{u,x_m \in [0,1]} |g^e(u, x_m)| \\
= &O_p(N_1^{-2}) + O_p(N_1/\sqrt{nT}) + O_p(h + (nh)^{-1/2}) \\
= &O_p\left((nT)^{-1/3} (\log nT) + n^{-1/3} \right).
\end{aligned}$$

This completes the proof. □

Proof of Theorem 2. The improved spline approximation of error process $\varepsilon_t(u)$ is given by

$$\hat{\varepsilon}_t(u) = \sum_{l=1}^{p} \sum_{r=1}^{N} \sum_{j=1}^{N} \hat{\mu}_{r,j,l} \int b_{r,j}(u, s) \hat{\varepsilon}_{t-l}(s) ds, \quad 0 \leq u \leq 1,$$

where the vector of coefficients $\hat{\boldsymbol{\mu}} = (\hat{\mu}_{1,1,1}, \cdots, \hat{\mu}_{N,N,p})^{\tau}$ is a pN^2-dimensional vector obtained by solving the following least squares problem:, i.e.,

$$\hat{\boldsymbol{\mu}} = \arg\min_{\boldsymbol{\mu}} \sum_{t=p+1}^{T} \sum_{i=1}^{n} \left[\tilde{\varepsilon}_t(u_i) - \sum_{l=1}^{p} \sum_{r=1}^{N} \sum_{j=1}^{N} \mu_{r,j,l} \int b_{r,j}(u_i, s) \tilde{\varepsilon}_{t-l}(s) ds \right]^2.$$

Define the cleaned signal as $f_t^c(u) = f_t(u) - \varepsilon_t(u)$, and its corresponding estimator $\hat{f}_t^c(u) = f_t(u) - \hat{\varepsilon}_t(u)$. Then, using a spline-based approach, the improved estimate of $g_m(u, x_m)$ takes the form

$$\hat{g}_m(u, x_m) = \sum_{r=1}^{N_0} \sum_{j=1}^{N_m} \hat{\lambda}_{r,j,m} b_{r,j,m}(u, x_m), \quad 1 \leq m \leq k,$$

where the coefficient vector $\hat{\boldsymbol{\lambda}} = (\hat{\lambda}_{1,1,1}, \cdots, \hat{\lambda}_{N_0,N_k,k})^T$ is a $(N_0 \sum_{m=1}^{k} N_m)$-dimensional vector minimizes the objective

$$\hat{\boldsymbol{\lambda}} = \arg\min_{\boldsymbol{\lambda}} \sum_{t=1}^{T} \sum_{i=1}^{n} \left[\hat{f}_t^c(u_i) - \sum_{m=1}^{k} z_{t,m} \sum_{r=1}^{N_0} \sum_{j=1}^{N_m} \lambda_{r,j,m} b_{r,j,m}(u_i, x_{t,m}) \right]^2.$$

Denote $\boldsymbol{f_t^c} = (f_t^c(u_1), \cdots, f_t^c(u_n))^\tau$ and $\boldsymbol{f^c} = (\boldsymbol{f_1^c}^\tau, \cdots, \boldsymbol{f_T^c}^\tau)^\tau$, and corresponding estimators $\hat{\boldsymbol{f}}_{\boldsymbol{t}}^{\boldsymbol{c}} = (\hat{f}_t^c(u_1), \cdots, \hat{f}_t^c(u_n))^\tau$ and $\hat{\boldsymbol{f}}^{\boldsymbol{c}} = (\hat{\boldsymbol{f}}_{\boldsymbol{1}}^{\boldsymbol{c}\,\tau}, \cdots, \hat{\boldsymbol{f}}_{\boldsymbol{T}}^{\boldsymbol{c}\,\tau})^\tau$. Furthermore, denote $\boldsymbol{e_t} = (e_t(u_1), \cdots, e_t(u_n))^\tau$ and $\boldsymbol{e} = (\boldsymbol{e_1}^\tau, \cdots, \boldsymbol{e_T}^\tau)^\tau$.

Given the estimate of $\boldsymbol{\varepsilon}$, the coefficient estimator of $\boldsymbol{\lambda}$ is given by

$$\hat{\boldsymbol{\lambda}} = (\boldsymbol{B}^\tau \boldsymbol{B})^{-1} \boldsymbol{B}^\tau \hat{\boldsymbol{f}}^{\boldsymbol{c}}, \quad \text{with} \quad \hat{\boldsymbol{f}}^{\boldsymbol{c}} = \boldsymbol{f} - \hat{\boldsymbol{\varepsilon}}.$$

However, due to density estimation error, $\hat{\boldsymbol{f}} = \boldsymbol{f} + \boldsymbol{\varepsilon_f}$, where $\boldsymbol{\varepsilon_f}$ is defined similarly as $\boldsymbol{\varepsilon}$, representing the transformation error. Then, define $\tilde{\hat{\boldsymbol{f}}}^{\boldsymbol{c}} = \hat{\boldsymbol{f}} - \hat{\boldsymbol{\varepsilon}}$, leading to the observation-based estimator of $\boldsymbol{\lambda}$

$$\hat{\boldsymbol{\lambda}} = (\boldsymbol{B}^\tau \boldsymbol{B})^{-1} \boldsymbol{B}^\tau \tilde{\hat{\boldsymbol{f}}}^{\boldsymbol{c}}.$$

Following the argument in Theorem 1, we decompose the estimation error as $\hat{g}_m(u, x_m) - g_m(u, x_m)$ as

$$\begin{aligned}
&\hat{g}_m(u, x_m) - g_m(u, x_m) \\
=&\boldsymbol{b}^\tau(u, x_m)\boldsymbol{A_m}(\boldsymbol{B}^\tau\boldsymbol{B})^{-1}\boldsymbol{B}^\tau\tilde{\hat{\boldsymbol{f}}}^{\boldsymbol{c}} - g_m(u, x_m) \\
=&\boldsymbol{b}^\tau(u, x_m)\boldsymbol{A_m}(\boldsymbol{B}^\tau\boldsymbol{B})^{-1}\boldsymbol{B}^\tau(\boldsymbol{f} - \hat{\boldsymbol{\varepsilon}}) + \boldsymbol{b}^\tau(u, x_m)\boldsymbol{A_m}(\boldsymbol{B}^\tau\boldsymbol{B})^{-1}\boldsymbol{B}^\tau\boldsymbol{\varepsilon_f} - g_m(u, x_m) \\
=&\boldsymbol{b}^\tau(u, x_m)\boldsymbol{A_m}(\boldsymbol{B}^\tau\boldsymbol{B})^{-1}\boldsymbol{B}^\tau\boldsymbol{g} - g_m(u, x_m) \\
&+ \boldsymbol{b}^\tau(u, x_m)\boldsymbol{A_m}(\boldsymbol{B}^\tau\boldsymbol{B})^{-1}\boldsymbol{B}^\tau(\boldsymbol{\varepsilon} - \hat{\boldsymbol{\varepsilon}}) + \boldsymbol{b}^\tau(u, x_m)\boldsymbol{A_m}(\boldsymbol{B}^\tau\boldsymbol{B})^{-1}\boldsymbol{B}^\tau\boldsymbol{\varepsilon_f} \\
=&g^B(u, x_m) + g^V(u, x_m) + g^e(u, x_m),
\end{aligned}$$

where

$$\begin{aligned}
g^B(u, x_m) &= \boldsymbol{b}^\tau(u, x_m)\boldsymbol{A_m}(\boldsymbol{B}^\tau\boldsymbol{B})^{-1}\boldsymbol{B}^\tau\boldsymbol{g} - g_m(u, x_m), \\
g^V(u, x_m) &= \boldsymbol{b}^\tau(u, x_m)\boldsymbol{A_m}(\boldsymbol{B}^\tau\boldsymbol{B})^{-1}\boldsymbol{B}^\tau(\boldsymbol{\varepsilon} - \hat{\boldsymbol{\varepsilon}}), \\
g^e(u, x_m) &= \boldsymbol{b}^\tau(u, x_m)\boldsymbol{A_m}(\boldsymbol{B}^\tau\boldsymbol{B})^{-1}\boldsymbol{B}^\tau\boldsymbol{\varepsilon_f}.
\end{aligned}$$

For the bias term $g^B(u, x_m)$,

$$\begin{aligned}
g^B(u, x_m) &= \boldsymbol{b}^\tau(u, x_m)\boldsymbol{A_m}(\boldsymbol{B}^\tau\boldsymbol{B})^{-1}\boldsymbol{B}^\tau\boldsymbol{g} - g_m(u, x_m) \\
&= \boldsymbol{b}^\tau(u, x_m)\boldsymbol{A_m}(\boldsymbol{B}^\tau\boldsymbol{B})^{-1}\boldsymbol{B}^\tau[\boldsymbol{g} - \boldsymbol{B\lambda}] + \left[\boldsymbol{b}^\tau(u, x_m)\boldsymbol{\lambda_m} - g_m(u, x_m)\right] \\
&= \boldsymbol{b}^\tau(u, x_m)\boldsymbol{A_m}(\frac{1}{nT}\boldsymbol{B}^\tau\boldsymbol{B})^{-1}\boldsymbol{B}^\tau\left[\frac{1}{nT}(\boldsymbol{g} - \boldsymbol{B\lambda})\right] + \left[\boldsymbol{b}^\tau(u, x_m)\boldsymbol{\lambda_m} - g_m(u, x_m)\right].
\end{aligned}$$

Utilizing the similar arguments as in Theorem 1 and leveraging spline approximation theory, we can have

$$\sup_{u, x_m \in [0,1]} |g^B(u, x_m)| = O_p(N_1^{-2}).$$

For the variance term $g^V(u, x_m) = \boldsymbol{b}^\tau(u, x_m)\boldsymbol{A_m}(\boldsymbol{B}^\tau\boldsymbol{B})^{-1}\boldsymbol{B}^\tau(\boldsymbol{\varepsilon} - \hat{\boldsymbol{\varepsilon}})$, express the autoregressive structure of the functional error process as

$$\varepsilon_t(u) = \sum_{l=1}^{p} \int \gamma_l(u, s)\varepsilon_{t-l}(s)ds + e_t(u) = \sum_{l=1}^{p}\sum_{r=1}^{N}\sum_{j=1}^{N} \mu_{r,j,l} \int b_{r,j}(u, s)\varepsilon_{t-l}(s)ds + e_t(u),$$

with the corresponding approximation

$$\hat{\varepsilon}_t(u) = \sum_{l=1}^{p}\sum_{r=1}^{N}\sum_{j=1}^{N}\hat{\mu}_{r,j,l}\int b_{r,j}(u,s)\tilde{\varepsilon}_{t-l}(s)ds, \quad 0 \le u \le 1.$$

Denote $\boldsymbol{b_t}(u) = (\int b_{1,1}(u,s)\tilde{\varepsilon}_{t-1}(s)ds, \ldots, \int b_{N,N}(u,s)\tilde{\varepsilon}_{t-p}(s)ds)^\tau$, $\boldsymbol{b_t} = (\boldsymbol{b_t}(u_1), \ldots, \boldsymbol{b_t}(u_n))^\tau$, and define the design matrix $\boldsymbol{B_\varepsilon} = (\boldsymbol{b_p^\tau}, \ldots, \boldsymbol{b_T^\tau})^\tau$. Furthermore, denote $\boldsymbol{\varepsilon_t} = (\varepsilon_t(u_1), \ldots, \varepsilon_t(u_n))^\tau$, $\boldsymbol{\varepsilon} = (\boldsymbol{\varepsilon_{p+1}^\tau}, \ldots, \boldsymbol{\varepsilon_T^\tau})^\tau$.

The model for the error process can be expressed in the form of matrix as $\boldsymbol{\varepsilon} \approx \boldsymbol{B_\varepsilon\mu} + \boldsymbol{e}$. Based on the initial estimates, we obtain a similarly structured model

$$\tilde{\boldsymbol{\varepsilon}} \approx \boldsymbol{B_\varepsilon\mu} + \tilde{\boldsymbol{e}},$$

where $\tilde{\boldsymbol{\varepsilon}}$ is is constructed analogously to $\boldsymbol{\varepsilon}$, and $\tilde{\boldsymbol{e}}$ denotes the residual based on the preliminary estimates.

The least squares estimator for $\boldsymbol{\mu}$ is given by

$$\hat{\boldsymbol{\mu}} = (\boldsymbol{B_\varepsilon^\tau B_\varepsilon})^{-1}\boldsymbol{B_\varepsilon^\tau}\tilde{\boldsymbol{\varepsilon}}.$$

Now consider the approximation error of the estimated error process:

$$\begin{aligned}\hat{\varepsilon}_t(u) - \varepsilon_t(u) &= \sum_{l=1}^{p}\sum_{r=1}^{N}\sum_{j=1}^{N}\hat{\mu}_{r,j,l}\int b_{r,j}(u,s)\tilde{\varepsilon}_{t-l}(s)ds - \varepsilon_t(u) \\ &= \boldsymbol{b_t^\tau}(u)(\boldsymbol{B_\varepsilon^\tau B_\varepsilon})^{-1}\boldsymbol{B_\varepsilon^\tau}\tilde{\boldsymbol{\varepsilon}} - \varepsilon_t(u) \\ &= \boldsymbol{b_t^\tau}(u)(\boldsymbol{B_\varepsilon^\tau B_\varepsilon})^{-1}\boldsymbol{B_\varepsilon^\tau}(\tilde{\boldsymbol{\varepsilon}} - \boldsymbol{B_\varepsilon\mu}) + (\boldsymbol{b_t^\tau}(u)\boldsymbol{\mu} - \varepsilon_t(u)).\end{aligned}$$

From spline approximation theory and under sufficient smoothness conditions, there exist a constant C_t, such that

$$\sup_u |\varepsilon_t(u) - \boldsymbol{b_t^\tau}(u)\boldsymbol{\mu}| \le C_t N^{-2}.$$

Moreover, by similar arguments, there exists a constant C_p such that

$$\sup_u |\tilde{\boldsymbol{\varepsilon}} - \boldsymbol{B_\varepsilon\mu}| \le C_p N^{-2}.$$

Combining the above, it follows that

$$\sup_u |\tilde{\boldsymbol{\varepsilon}} - \boldsymbol{\varepsilon}| = O_p(N^{-2}).$$

Therefore, the second term in the decomposition of the total estimation error satisfies

$$\sup_{u,x_m\in[0,1]} |g^V(u,x_m)| = \sup_{u,x_m\in[0,1]} |\boldsymbol{b}^\tau(u,x_m)\boldsymbol{A_m}(\boldsymbol{B}^\tau\boldsymbol{B})^{-1}\boldsymbol{B}^\tau(\varepsilon - \hat{\varepsilon})| = O_p(N^{-2}).$$

Consider now the third component of the error $g^e(u,x_m) = \boldsymbol{b}^\tau(u,x_m)\boldsymbol{A_m}(\boldsymbol{B}^\tau\boldsymbol{B})^{-1}\boldsymbol{B}^\tau\boldsymbol{\varepsilon_f}$, which arises from the kernel density estimation of $f_t(u)$. Under the smoothness conditions for the function f_t, and applying established results from kernel smoothing theory (as in the proof of Theorem 1, we can get that

$$\sup_{u,x_m\in[0,1]} |g^e(u,x_m)| = O_p(h + (nh)^{-1/2}).$$

Therefore, assuming $h \sim n^{-1/3}$, $N_0, N_m \sim (nT)^{1/6}\log nT$, namely, $N_1 \sim (nT)^{1/6}\log nT$, $N \sim (nT)^{1/6}\log nT$, as $n, T \to \infty$, we derive the overall convergence rate of the estimator as

$$\begin{aligned}
&\sup_{u,x_m\in[0,1]} |\hat{g}_m(u,x_m) - g_m(u,x_m)| \\
\le &\sup_{u,x_m\in[0,1]} |g^B(u,x_m)| + \sup_{u,x_m\in[0,1]} |g^V(u,x_m)| + \sup_{u,x_m\in[0,1]} |g^e(u,x_m)| \\
= &O_p(N_1^{-2}) + O_p(N^{-2}) + O_p(N_1^{-2} + h + (nh)^{-1/2}) \\
= &O_p\left((nT)^{-1/3}(\log nT)^{-2} + n^{-1/3}\right).
\end{aligned}$$

This completes the proof. □

Proof of Theorem 3. (i) We first establish the asymptotic normality of the initial estimatior $\tilde{g}_m(u,x_m)$. Leveraging assumptions (A1)-(A4), we can express the centered and scaled estimation error as $\sqrt{nT}(\tilde{g}_m(u,x_m) - g_m(u,x_m))$ as

$$\begin{aligned}
&\sqrt{nT}(\tilde{g}_m(u,x_m) - g_m(u,x_m)) \\
= &\sqrt{nT}[\boldsymbol{b}^\tau(u,x_m)\boldsymbol{A_m}(\boldsymbol{B}^\tau\boldsymbol{B})^{-1}\boldsymbol{B}^\tau\boldsymbol{g} - g_m(u,x_m)] \\
&+ \sqrt{nT}[\boldsymbol{b}^\tau(u,x_m)\boldsymbol{A_m}(\boldsymbol{B}^\tau\boldsymbol{B})^{-1}\boldsymbol{B}^\tau\boldsymbol{\varepsilon} + \boldsymbol{b}^\tau(u,x_m)\boldsymbol{A_m}(\boldsymbol{B}^\tau\boldsymbol{B})^{-1}\boldsymbol{B}^\tau\boldsymbol{\varepsilon_f}].
\end{aligned}$$

Since the error process $\boldsymbol{\varepsilon_t}$ is independent of the covariates $\boldsymbol{x_t}, \boldsymbol{z_t}$, we have $E(\boldsymbol{\varepsilon_t}|\boldsymbol{x_t}, \boldsymbol{z_t}) = 0$. Together with the result established in Theorem 1, it follows that:

$$\begin{aligned}
&E[\sqrt{nT}(\tilde{g}_m(u,x_m) - g_m(u,x_m))] \\
= &\sqrt{nT}E[\boldsymbol{b}^\tau(u,x_m)\boldsymbol{A_m}(\boldsymbol{B}^\tau\boldsymbol{B})^{-1}\boldsymbol{B}^\tau(\boldsymbol{g} - \boldsymbol{B\lambda})] + \sqrt{nT}E[\boldsymbol{b}^\tau(u,x_m)\boldsymbol{\lambda_m} - g_m(u,x_m)] \\
&+ \sqrt{nT}E[\boldsymbol{b}^\tau(u,x_m)\boldsymbol{A_m}(\boldsymbol{B}^\tau\boldsymbol{B})^{-1}\boldsymbol{B}^\tau E(\boldsymbol{\varepsilon}|\boldsymbol{x},\boldsymbol{z})] \\
&+ \sqrt{nT}E[\boldsymbol{b}^\tau(u,x_m)\boldsymbol{A_m}(\boldsymbol{B}^\tau\boldsymbol{B})^{-1}\boldsymbol{B}^\tau E(\boldsymbol{\varepsilon_f}|\boldsymbol{x},\boldsymbol{z})] \\
= &0.
\end{aligned}$$

Next, we compute the variance

$$\begin{aligned}
&Var[\sqrt{nT}(\tilde{g}_m(u,x_m) - g_m(u,x_m))] \\
= &nTVar[\boldsymbol{b}^\tau(u,x_m)\boldsymbol{A_m}(\boldsymbol{B}^\tau\boldsymbol{B})^{-1}\boldsymbol{B}^\tau\boldsymbol{\varepsilon} + \boldsymbol{b}^\tau(u,x_m)\boldsymbol{A_m}(\boldsymbol{B}^\tau\boldsymbol{B})^{-1}\boldsymbol{B}^\tau\boldsymbol{\varepsilon_f}]
\end{aligned}$$

According to the results shown in Theorem 1, we can get that

$$\begin{aligned}
&nTVar[\boldsymbol{b}^\tau(u,x_m)\boldsymbol{A_m}(\boldsymbol{B}^\tau\boldsymbol{B})^{-1}\boldsymbol{B}^\tau\boldsymbol{\varepsilon}] \\
= &nTE\left[E\left(\boldsymbol{b}^\tau(u,x_m)\boldsymbol{A_m}(\boldsymbol{B}^\tau\boldsymbol{B})^{-1}\boldsymbol{B}^\tau\boldsymbol{\varepsilon}|\boldsymbol{x},\boldsymbol{z}\right)^2\right] \\
= &nTE\left[E\left(\boldsymbol{b}^\tau(u,x_m)\boldsymbol{A_m}(\boldsymbol{B}^\tau\boldsymbol{B})^{-1}\boldsymbol{B}^\tau\boldsymbol{\varepsilon}\boldsymbol{\varepsilon}^\tau\boldsymbol{B}(\boldsymbol{B}^\tau\boldsymbol{B})^{-1}\boldsymbol{A}_{\boldsymbol{m}}^\tau\boldsymbol{b}(u,x_m)|\boldsymbol{x},\boldsymbol{z}\right)\right].
\end{aligned}$$

For notational simplicity, define $\boldsymbol{B_*} = \boldsymbol{B}/\sqrt{nT}$, and denote $\boldsymbol{D_m} = \boldsymbol{A_m}(\boldsymbol{B_*}^\tau\boldsymbol{B_*})^{-1}\boldsymbol{B_*}^\tau$. Then the variance component simplifies to

$$nTVar[\boldsymbol{b}^\tau(u,x_m)\boldsymbol{A_m}(\boldsymbol{B}^\tau\boldsymbol{B})^{-1}\boldsymbol{B}^\tau\boldsymbol{\varepsilon}] = E\left[\boldsymbol{b}^\tau(u,x_m)E\left(\boldsymbol{D_m}\boldsymbol{\varepsilon}\boldsymbol{\varepsilon}^\tau\boldsymbol{D}_{\boldsymbol{m}}^\tau|\boldsymbol{x},\boldsymbol{z}\right)\boldsymbol{b}^\tau(u,x_m)\right].$$

Denote $\boldsymbol{\Sigma}_\varepsilon = E(\boldsymbol{\varepsilon}\boldsymbol{\varepsilon}^\tau|\boldsymbol{x}, z)$, given the auto-regressive nature of the error process, the covariance matrix can be decomposed into two parts, namely, $\boldsymbol{\Sigma}_\varepsilon = \boldsymbol{\Sigma}_1 + \boldsymbol{\Sigma}_2$, where $\boldsymbol{\Sigma}_1 = \text{diag}(\boldsymbol{\Sigma}_{t,t})_{1\le t\le T}$ captures the within-time variance structure, with $\boldsymbol{\Sigma}_{t,t} = Cov(\boldsymbol{\varepsilon}_t)$, and $\boldsymbol{\Sigma}_2$ contains the off-diagonal elements as $\boldsymbol{\Sigma}_2 = (\boldsymbol{\Sigma}_{i,j})_{1\le t\ne s\le T} = (Cov(\boldsymbol{\varepsilon}_t, \boldsymbol{\varepsilon}_s))_{1\le t\ne s\le T}$, account for temporal dependence among errors across time points.

Similarly, for the functional estimation error $\boldsymbol{\varepsilon}_f$, we have

$$\begin{aligned}
&nTVar[\boldsymbol{b}^\tau(u, x_m)\boldsymbol{A}_m(\boldsymbol{B}^\tau\boldsymbol{B})^{-1}\boldsymbol{B}^\tau\boldsymbol{\varepsilon}_f] \\
=&nTE\left[E\left(\boldsymbol{b}^\tau(u, x_m)\boldsymbol{A}_m(\boldsymbol{B}^\tau\boldsymbol{B})^{-1}\boldsymbol{B}^\tau\boldsymbol{\varepsilon}_f\boldsymbol{\varepsilon}_f^\tau\boldsymbol{B}(\boldsymbol{B}^\tau\boldsymbol{B})^{-1}\boldsymbol{A}_m^\tau\boldsymbol{b}(u, x_m)|\boldsymbol{x}, \boldsymbol{z}\right)\right] \\
=&nT\sigma_\epsilon^2 E\left[E\left(\boldsymbol{b}^\tau(u, x_m)\boldsymbol{A}_m(\boldsymbol{B}^\tau\boldsymbol{B})^{-1}\boldsymbol{A}_m^\tau\boldsymbol{b}(u, x_m)|\boldsymbol{x}, \boldsymbol{z}\right)\right].
\end{aligned}$$

Under regularity conditions, and due to the smoothness of the underlying functional process, this variance component tends to zero as $n, T \to \infty$.

Therefore, applying the Linderberg-Feller central limit theorem, justified by the boundedness of second moments as established in Theorem 1, and under the asymptotic regime $n \gg T \to \infty$, we conclude that

$$\sqrt{nT}(\boldsymbol{C}_m\boldsymbol{\Sigma}_\varepsilon\boldsymbol{C}_m^\tau)^{-1}(\tilde{g}_m(u, x_m) - g_m(u, x_m)) \xrightarrow{D} N(0, 1),$$

where $\boldsymbol{C}_m = \boldsymbol{b}^\tau(u, x_m)E(\boldsymbol{D}_m) = \boldsymbol{b}^\tau(u, x_m)E(\boldsymbol{A}_m(\boldsymbol{B}_*^\tau\boldsymbol{B}_*)^{-1}\boldsymbol{B}_*^\tau)$, and $\boldsymbol{\Sigma}_\varepsilon = (\boldsymbol{\Sigma}_{t,s})_{1\le t,s\le T}$ with $\boldsymbol{\Sigma}_{t,s} = Cov(\boldsymbol{\varepsilon}_t, \boldsymbol{\varepsilon}_s)$ representing the temporal dependence structure of the error process.

(ii) After obtaining the spline-based estimator of the error process $\hat{\boldsymbol{\varepsilon}}$, we refine the estimation of bivariate varying-coefficient functions $g_m(u, x_m)$ based on the adjusted model

$$f_t^c(u) = f_t(u) - \varepsilon_t(u) = \sum_{m=1}^{k} z_{t,m}g_m(u, x_{t,m}) + e_t(u),$$

where $e_t(u)$ represents the residual error after removing the estimated error process.

As established in Theorem 1, the estimation error for the refined estimator can be expressed as

$$\begin{aligned}
&\sqrt{nT}(\hat{g}_m(u, x_m) - g_m(u, x_m)) \\
=&\sqrt{nT}[\boldsymbol{b}^\tau(u, x_m)\boldsymbol{A}_m(\boldsymbol{B}^\tau\boldsymbol{B})^{-1}\boldsymbol{B}^\tau\tilde{\boldsymbol{f}}^c - g_m(u, x_m)] \\
=&\sqrt{nT}[\boldsymbol{b}^\tau(u, x_m)\boldsymbol{A}_m(\boldsymbol{B}^\tau\boldsymbol{B})^{-1}\boldsymbol{B}^\tau(\boldsymbol{f} - \hat{\boldsymbol{\varepsilon}}) + \boldsymbol{b}^\tau(u, x_m)\boldsymbol{A}_m(\boldsymbol{B}^\tau\boldsymbol{B})^{-1}\boldsymbol{B}^\tau\boldsymbol{\varepsilon}_f - g_m(u, x_m)] \\
=&\sqrt{nT}[\boldsymbol{b}^\tau(u, x_m)\boldsymbol{A}_m(\boldsymbol{B}^\tau\boldsymbol{B})^{-1}\boldsymbol{B}^\tau\boldsymbol{g} - g_m(u, x_m)] \\
&+ \sqrt{nT}[\boldsymbol{b}^\tau(u, x_m)\boldsymbol{A}_m(\boldsymbol{B}^\tau\boldsymbol{B})^{-1}\boldsymbol{B}^\tau(\boldsymbol{\varepsilon} - \hat{\boldsymbol{\varepsilon}}) + \boldsymbol{b}^\tau(u, x_m)\boldsymbol{A}_m(\boldsymbol{B}^\tau\boldsymbol{B})^{-1}\boldsymbol{B}^\tau\boldsymbol{\varepsilon}_f].
\end{aligned}$$

Since $\hat{\boldsymbol{\varepsilon}}$ is a consistent estimator of the error process $\boldsymbol{\varepsilon}$, it follows from the convergence results established in Theorem 2 that

$$E(\sqrt{nT}[\tilde{g}_m(u, x_m) - g_m(u, x_m)] = 0.$$

Next, consider the variance of the refined estimator

$$\begin{aligned}
&Var[\sqrt{nT}(\hat{g}_m(u, x_m) - g_m(u, x_m))] \\
=&nTVar[\boldsymbol{b}^\tau(u, x_m)\boldsymbol{A}_m(\boldsymbol{B}^\tau\boldsymbol{B})^{-1}\boldsymbol{B}^\tau(\boldsymbol{g} + \boldsymbol{\varepsilon} - \hat{\boldsymbol{\varepsilon}} - \boldsymbol{\varepsilon}_f) - g_m(u, x_m)] \\
=&nTVar[\boldsymbol{b}^\tau(u, x_m)\boldsymbol{A}_m(\boldsymbol{B}^\tau\boldsymbol{B})^{-1}\boldsymbol{B}^\tau(\boldsymbol{\varepsilon} - \hat{\boldsymbol{\varepsilon}}) + \boldsymbol{b}^\tau(u, x_m)\boldsymbol{A}_m(\boldsymbol{B}^\tau\boldsymbol{B})^{-1}\boldsymbol{B}^\tau\boldsymbol{\varepsilon}_f].
\end{aligned}$$

By applying the same arguments as in the first part and invoking the law of total variance, we have

$$
\begin{aligned}
& nTVar[\boldsymbol{b}^\tau(u,x_m)\boldsymbol{A_m}(\boldsymbol{B}^\tau\boldsymbol{B})^{-1}\boldsymbol{B}^\tau(\boldsymbol{\varepsilon}-\hat{\boldsymbol{\varepsilon}})] \\
=& nTE\left[E\left(\boldsymbol{b}^\tau(u,x_m)\boldsymbol{A_m}(\boldsymbol{B}^\tau\boldsymbol{B})^{-1}\boldsymbol{B}^\tau(\boldsymbol{\varepsilon}-\hat{\boldsymbol{\varepsilon}})|\boldsymbol{x},\boldsymbol{z}\right)^2\right] \\
=& nTE\left[E\left(\boldsymbol{b}^\tau(u,x_m)\boldsymbol{A_m}(\boldsymbol{B}^\tau\boldsymbol{B})^{-1}\boldsymbol{B}^\tau(\boldsymbol{\varepsilon}-\hat{\boldsymbol{\varepsilon}})(\boldsymbol{\varepsilon}-\hat{\boldsymbol{\varepsilon}})^\tau\boldsymbol{B}(\boldsymbol{B}^\tau\boldsymbol{B})^{-1}\boldsymbol{A}^\tau_{\boldsymbol{m}}\boldsymbol{b}(u,x_m)|\boldsymbol{x},\boldsymbol{z}\right)\right] \\
=& E\left[\boldsymbol{b}^\tau(u,x_m)\boldsymbol{D_m}E((\boldsymbol{\varepsilon}-\hat{\boldsymbol{\varepsilon}})(\boldsymbol{\varepsilon}-\hat{\boldsymbol{\varepsilon}})^\tau|\boldsymbol{x},\boldsymbol{z})\boldsymbol{D}^\tau_{\boldsymbol{m}}\boldsymbol{b}(u,x_m)\right],
\end{aligned}
$$

where we denote $\boldsymbol{D_m}=\boldsymbol{A_m}(\boldsymbol{B}^\tau\boldsymbol{B})^{-1}$.

Denote the covariance matrix $\boldsymbol{\Xi_\varepsilon}=E((\boldsymbol{\varepsilon}-\hat{\boldsymbol{\varepsilon}})(\boldsymbol{\varepsilon}-\hat{\boldsymbol{\varepsilon}})^\tau|\boldsymbol{x},\boldsymbol{z})$. similarly, it can also be decomposed into two parts as $\boldsymbol{\Xi_\varepsilon}=\boldsymbol{\Xi_1}+\boldsymbol{\Xi_2}$, where $\boldsymbol{\Xi_1}=\mathrm{diag}(\boldsymbol{\Xi_{t,t}})_{1\le t\le T}$ is a block diagnoal matrix with the t-th diagonal block given by $\boldsymbol{\Xi_{t,t}}=Cov(\boldsymbol{\varepsilon_t}-\hat{\boldsymbol{\varepsilon}}_{\boldsymbol{t}})$, and $\boldsymbol{\Xi_2}$ contains the off-diagonal blocks as $\boldsymbol{\Xi_2}=(\boldsymbol{\Xi_{t,s}})_{1\le t\ne s\le T}=(Cov(\boldsymbol{\varepsilon_t}-\hat{\boldsymbol{\varepsilon}}_{\boldsymbol{t}},\boldsymbol{\varepsilon_s}-\hat{\boldsymbol{\varepsilon}}_{\boldsymbol{s}}))_{1\le t\ne s\le T}$.

Due to the consistency and convergence properties of the estimator $\boldsymbol{\varepsilon}-\hat{\boldsymbol{\varepsilon}}$, as $n,T\to\infty$, the off-diagonal covariance terms vanish. Consequently, the covariance matrix reduces to $\boldsymbol{\Xi_\varepsilon}=\boldsymbol{\Xi_1}=Cov(\boldsymbol{e_t})$. Moreover, since the residual covariance $Cov(e_t(u),e_t(s))=\sigma_t^2(u,s)$ characterizes the within-time covariance structure, we have $\boldsymbol{\Xi_\varepsilon}=Cov(\boldsymbol{e_t})=\mathrm{diag}(\boldsymbol{\Xi_{t,t}})_{1\le t\le T}$, where $\boldsymbol{\Xi_{t,t}}(u,s)=\sigma_t^2(u,s)$.

Therefore, the asymptotic distribution of the refined estimator satisfies

$$
\sqrt{nT}(\boldsymbol{C_m}\boldsymbol{\Xi_\varepsilon}\boldsymbol{C}^\tau_{\boldsymbol{m}})^{-1}(\hat{g}_m(u,x_m)-g_m(u,x_m))\xrightarrow{D}N(0,1),
$$

where $\boldsymbol{C_m}=\boldsymbol{b}^\tau(u,x_m)E(\boldsymbol{A_m}(\boldsymbol{B}^\tau_*\boldsymbol{B}_*)^{-1}\boldsymbol{B}^\tau_*)$, and $\boldsymbol{\Xi_\varepsilon}=\mathrm{diag}(\boldsymbol{\Xi_{t,t}})_{1\le t\le T}$ with $\boldsymbol{\Xi_{t,t}}(u,s)=\sigma_t^2(u,s)$.

This completes the proof of the theorem. □

References

1. Stone, C. The use of polynomial splines and their tensor products in multivariate function estimation. *The Annals of Statistics* **1994**, *22*, 118–171.
2. DeVore, R.; Lorentz, G. *Constructive Approximation, Volume 303*; Springer Science & Business Media: New York, NY, USA, 1993.
3. Petersen, A.; Müller, H. Functional data analysis for density functions by transformation to a Hilbert space. *The Annals of Statisics* **2016**, *44*, 183–218.

Disclaimer/Publisher's Note: The statements, opinions and data contained in all publications are solely those of the individual author(s) and contributor(s) and not of MDPI and/or the editor(s). MDPI and/or the editor(s) disclaim responsibility for any injury to people or property resulting from any ideas, methods, instructions or products referred to in the content.
