# Supplementary figures and images for: Varying-Coefficient Additive Models with Density Responses and Functional Auto-Regressive Error Process
Source: Entropy (Basel). 2025 Aug 20;27(8):882. doi: 10.3390/e27080882 (PMC12385778; doi:10.3390/e27080882)

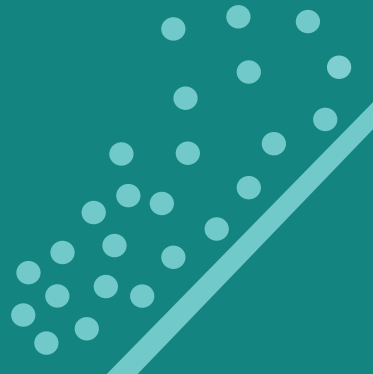

*entropy*

Supplement: Supplementary file 1 [file entropy-27-00882-s001.zip › Definitions/entropy-logo-eps-converted-to.pdf]

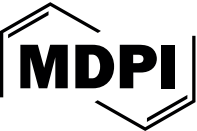

Supplement: Supplementary file 1 [file entropy-27-00882-s001.zip › Definitions/logo-mdpi-eps-converted-to.pdf]

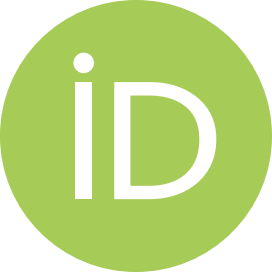

Supplement: Supplementary file 1 [file entropy-27-00882-s001.zip › Definitions/logo-orcid.pdf]

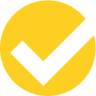

check for  
updates

Supplement: Supplementary file 1 [file entropy-27-00882-s001.zip › Definitions/logo-updates-eps-converted-to.pdf]
